# Mental health symptoms in Chinese children with sleep disorders and association with parental emotions

**DOI:** 10.1038/s41598-025-14305-4

**Published:** 2025-08-26

**Authors:** Changqing Sun, Wei Xue, Peijia Zhang, Zhengqi Zhu, Bo Hu, Panpan Wang, Yang Li, Mingyang Zhao, Jiong Hao, Qianyu Zhou, Dequan Sun, Jiale Han, Zizheng Liu

**Affiliations:** 1 School of Nursing and Health, Zhengzhou University, 101 Kexue Avenue, Zhengzhou, Henan 450001 China; 2 College of Public Health, Zhengzhou University, Zhengzhou, Henan 450001 China; 3 College of Nursing, University of Manitoba, Winnipeg, MB R3T 2N2 Canada; 4 Department of Psychosomatic Disorders, Haining Fourth People’s Hospital, Jiaxing, Zhejiang 314400 China

**Keywords:** Epidemiology, Psychology

## Abstract

Depression, anxiety, and stress are major mental health challenges during children’s development. In China, heightened sleep disorders (SD), parental expectations, and academic stress highlight the importance of this issue. A total of 6,635 SD child-parent dyads were included in analysis. Sleep quality and psychological variables were assessed using Pittsburgh Sleep Quality Index (PSQI), Depression Anxiety Stress Scales (DASS) and Satisfaction with Life Scale (SWLS). Children with SD exhibited significantly higher rates of depression, anxiety, stress and life dissatisfaction. We identified the core and bridge symptoms within the Chinese children mental health symptoms and explored their associations with parental mental health. Worthlessness and palpitations, exhibiting the highest centrality values, were identified as core symptoms in the SD group. Palpitations were associated with elevated parental anxiety and stress. Impending collapse associated with parental anxiety, emerging as a bridge symptom may aggravate the occurrence of depression, anxiety and stress comorbidity in children. These symptoms provide theoretical basis for the targeted psychological intervention of Chinese children with SD in the future.

**Supplementary Information:**

The online version contains supplementary material available at 10.1038/s41598-025-14305-4.

## Introduction

Sleep disorders (SD) negatively impacts the physical, mental, and social health of children and adolescents^1,2^. Poor sleep quality is known to amplify negative emotions, potentially contributing to mental health issues^1,3,4^. In China, the high prevalence of SD among this age group is frequently disregarded, resulting in many cases being left untreated^5–8^. The overall prevalence of sleep disturbances was 26.0% in children and adolescents^5,9^. The 2021 UNICEF World Children Report highlighted a significant rise in mental health disorders among minors, marking a substantial increase over nearly three decades and now ranking as the third leading cause of childhood disability and mortality^10^. Depression, anxiety, and stress are major mental health challenges during children’s development, particularly as they form their self-identity^8,11–13^.

Disrupted sleep patterns can lead to hormonal imbalances, particularly affecting cortisol levels, which are crucial for regulating the body’s circadian rhythm^2^. Hormonal disruptions can impair cognitive functions such as memory and concentration. The biopsychosocial model proposes that elevated hormone levels, a common issue among those susceptible to anxiety and depression, can increase physiological arousal^14,15^. Disrupted sleep patterns can lead to hormonal imbalances, particularly affecting cortisol (a stress hormone regulating circadian rhythms) and melatonin (a sleep-promoting hormone)^2,4^. Elevated cortisol levels, often seen in anxious or depressed individuals, increase physiological arousal, while reduced melatonin impairs sleep quality, creating a bidirectional loop where poor sleep and hormonal dysregulation exacerbate mental health symptoms^15,16^. This heightened arousal state can exacerbate sleep problems, including insomnia, frequent awakenings, or insufficient sleep^1^. These disruptions intensify mental distress and disrupt daily life, perpetuating a cycle that hinders recovery^1,2^.

The link between parental metal issues in infancy and lasting emotional challenges in children is significant^17^. These effects continue to manifest even after accounting for changes in risk factors. The transition to adolescence is particularly critical, as it is a vulnerable period for the development of symptoms in children^18^. Borsboom’s psychopathology network theory (PNT) posits that external events can trigger specific internal symptoms^19^. Such as chronic sleep deprivation from external pressures, parental expectations and excessive academic stress are common stressors among Chinese youth, can trigger specific internal symptoms like palpitations (somatic arousal) or social anxiety (fear of academic evaluation)^19,20^ These and other symptoms then generate a stable and continuous feedback loop, for instance, palpitations may heighten anxiety about falling asleep, worsening sleep quality and reinforcing both somatic and emotional distress^1,21^that eventually leads to the dynamic development and maintenance of the mental disorder^19^. Evidence from previous studies, such as brain imaging^16^cortisol^2^ and β-amyloid^4^has confirmed that sleep disturbances are associated with mental health issues. Recognizing the characteristic symptoms is essential for preventing mental health disorders that occur early.

Traditional statistical approaches, such as t-tests, ANOVA, or regression models, excel at evaluating pairwise associations or group differences in aggregate symptom scores (e.g., total depression, anxiety, or stress scores). However, these methods often overlook the dynamic interdependencies between individual symptoms, which are central to understanding how mental health issues emerge and persist^21,22^. For example, a child with sleep disorders may experience not just elevated anxiety overall but specific symptom interactions, such as how social anxiety intensifies feelings of unease, which in turn disrupts sleep further-a nuance lost in aggregate scores. Network analysis (NA), rooted in PNT^19,22^visualizes symptoms as “nodes” and their statistical associations (estimated via Gaussian Graphical Models and Absolute Shrinkage and Selection Operator) as “edges,” revealing how symptoms activate, amplify, or sustain each other^23^. Edges represent direct relationships between symptoms, controlling all other symptoms in the network. The link between sleep quality and trouble relaxing remained strong even after accounting for other emotional states^24,25^. Unlike conventional techniques that treat symptoms as independent variables, NA captures complex, reciprocal relationships. This is critical for identifying core symptoms, which exert the strongest influence on the network, and bridge symptoms, which link distinct symptom clusters and facilitate comorbidity, both serving as high-impact targets for intervention^26^. For instance, somatic complaint may disrupt multiple anxiety and depressive symptoms simultaneously^27^, this hypothesis difficult to test with traditional methods focused on group means. Additionally, NA enables group-specific network comparisons (e.g., sleep disorders vs. non-sleep disorders), revealing structural differences in symptom connectivity^24^. Conventional approaches, by contrast, would only report higher anxiety prevalence in the sleep disorders group but not the unique symptom interactions that drive this disparity. Using propensity score matching (PSM) to control for demographic confounders (age, gender, region, school stage, and only-child status), comparing mental health symptom and association with parental emotions between children with and without sleep disorders, would help identify individualized intervention targets^28,29^. Integrating these methods can not only balance confounding variables but also explore how the mental health symptom network itself adapts to sleep disruption and parental emotional influnces.

Our objectives: (a) compare the prevalence of depression, anxiety, stress, life satisfaction, and parental negative emotions between children with and without sleep disorders; (b) examine and contrast the network structure of these symptoms to identify core, bridge, and strongly connected symptoms in relation to parental emotions; and (c) assess group differences in network topology and explore associations with parental mental health to inform targeted early interventions.

## Methods

### Participants and measurement

This study utilized a regional sample from the central plains, China, a demographically diverse area with urban, town, and village populations. Schools were recruited through partnerships with local education bureaus, stratified by urbanicity to ensure regional representativeness. Surveys were administered in-person during school hours by trained researchers, with completed digitally via tablets and on paper for schools lacking internet access. Before conducting the survey, all investigators received standardized training to ensure consistency in data collection procedures. In accordance with ethical guidelines for research involving minors, informed consent was obtained from all legal guardians, and assent was secured from participating children. Trained investigators were available to clarify any questionnaire items as needed, ensuring participants fully understood the questions without influencing their responses. The minimum time to respond to the questionnaire was 10 min, and if the answer was too fast or selected the same option consecutively, the sample would be treated as invalid. A screening question “Which one is not an animal?” was included in the survey. Two investigators concurrently reviewed the data and checked for obvious errors and data inconsistencies. All researchers involved held at least a medical master’s degree and participated in at least two similar research projects. All methods were conducted in accordance with the ethical principles of the Declaration of Helsinki and relevant guidelines for research involving human participants. The study protocol was reviewed and approved by the Ethics Committee of Zhengzhou University (Approval No. ZZUIRB2022-17).

Participants were selected from 157 districts/counties across 18 cities in the central part of China. These districts/counties were divided into four layers based on their total GDP, ranging from high to low^30^. From each layer, four districts/counties were randomly selected as the primary sampling units. These sampled districts/counties were then stratified by urban (street) and rural (town, village) areas^30^. Two streets/towns/villages were randomly selected from each district/county to form the secondary sampling units. Following this, 1–2 schools were chosen from each selected street/township using random sampling method. Within each school, 1–4 classes from each grade were randomly selected, and all students in the selected classes were included as research participants.

The demographic data according to their location is shown in Table S1. From March to July 2022, we distributed a total of 57,193 questionnaires to students, with consent obtained from their primary guardians. Some schools chose not to participate due to administrative constraints, but we found no systematic bias in the demographics of the non-respondents. After excluding invalid samples based on criteria such as missing data (more than 20%), duplicates, or responses to trap questions, we obtained 54,488 valid responses, resulting in a validity rate of 95.3%. Additionally, 50,536 parents completed the follow-up depression, anxiety, and stress scales, including 3,952 parents who participated twice (both of their own children were included in the study). Furthermore, 7,187 class teachers also contributed to the survey. This research aims to provide a more comprehensive understanding of the mental health issues facing Chinese children. This study was reviewed and approved by the Ethics Committee, and all participants and their parents signed consent forms.

The Pittsburgh Sleep Quality Index (PSQI) was employed to assess sleep quality^31^. It is the most widely used sleep assessment tool, including seven dimensions: subjective sleep quality, sleep latency, sleep duration, sleep efficiency, sleep disturbances, use of sleep medication, and daytime dysfunction^31,32^. Each component of the PSQI is scored on a scale from 0 to 3, with a score of 3 representing the highest level of dysfunction or disturbance. The total PSQI score is calculated by summing the seven component scores, resulting in a global score that ranges from 0 to 21. Higher total scores indicate worse sleep quality, and a score above 5 suggests the presence of significant sleep disturbances^31,33^. Psychological variables were assessed using the Depression Anxiety Stress Scales for youth (DASS-Y) 21 items^34,35^ and Satisfaction with Life Scale (SWLS)^36,37^. DASS-Y is known for its internal consistency and temporal stability, making it a reliable tool for children and adolescents^38–40^. Assessment of depressive symptoms encompassed 7 items concerning pathological dysthymia, low self-esteem, and a low level of positive emotion. A score of ≤ 9 was regarded as normal. Anxiety symptoms comprised 7 items related to the physical and subjective experience of anxiety arousal, with a score of ≤ 7 being defined as normal. Stress symptoms consisted of 7 items linked to negative emotions like tension, worry, and conflict, among others. A score of ≤ 4 was considered normal. Cut-off scores for clinical levels of depression, anxiety, and stress were derived from the DASS manual, defines thresholds based on symptom severity distributions in youth populations^34,40^. Parental mental health was assessed using the adult version of the 21-item Depression Anxiety Stress Scales (DASS-21)^34,35^. The SWLS is a short 5-item instrument designed to measure global cognitive judgments of satisfaction with one’s life. Each question is rated on a scale from 1 to 7, with a total score of 20 serving as the cut-off point. A score at this level indicates neutral life satisfaction, above which indicates satisfaction and below which indicates dissatisfaction. The scale usually requires only about one minute of a respondent’s time. Cronbach’s α (PSQI: 0.848, DASS: 0.952, SWLS: 0.929) and test-retest reliability (*r* = 0.82–0.89, *P* < 0.001) were excellent. Construct validity was supported by confirmatory factor analysis (CFI > 0.90, RMSEA < 0.08 for both scales).

### Data analysis

All statistical analyses were performed using the R-Studio program (version 4.3.2). Missing data were handled using multiple imputations via the *MICE* (Version 3.15.0) in R, with 20 iterations under the missing-at-random (MAR) assumption. This approach preserved the full sample size and minimized bias in statistical power. Outliers in continuous variables (e.g., sleep duration) were identified using Z-scores ( > ± 3) and winsorized to the 99th/1st percentiles to mitigate skew. Double-entry verification was conducted for 10% of surveys to ensure accuracy, with a discrepancy rate < 0.5% resolved by cross-checking original records. Given the continuous nature of variables, we employed the Pairwise Markov Random Field (PMRF) framework to estimate a Gaussian Graphical Model (GGM), which is well-suited for modeling partial correlations in multivariate continuous data^41,42^. PMRF ensures that each edge (symptom association) is estimated while controlling for all other symptoms, providing a sparse, interpretable network structure^22^. Normality of continuous variables was assessed via Shapiro-Wilk tests (*P* > 0.05). Multicollinearity was tested via variance inflation factor (VIF < 2.0 for all predictors). To ensure the reliability of the network analysis model, we used the *powerly* (version 1.8.6) package based on the Monte Carlo method to determine the sample size for the GGM with 21 network nodes^43^. It was calculated that a sample of at least 1780 samples was required to achieve acceptable statistical power (1- β = 0.8, sensitivity = 0.6). 6,654 out of 54,488 participants detected SD in Table 1, already enough for the network analysis model. Before constructing the networks, potential item redundancy was checked using the goldbricker function from the R package *networktools* (version 1.5.2). Following Jones’ manual^44^, if the proportion of significantly different central correlations between two variables and other items is less than 25%, then it can be confirmed that these two items measure the same trait or symptom (i.e., redundancy). Goldbricker analysis identified no item redundancy (all redundancy indices < 0.2), confirming that symptoms were conceptually distinct and suitable for network modeling. PSM was conducted using the R package *MatchIt* (version 4.5.5) to achieve 1:1 matching between children with and without SD^29^. The PSM were calculated through a multivariable logistic regression that included the following covariates: age, gender, region, school stage, and whether the child was an only child^29^. The caliper value was set at 0.01, using the *Nearest Neighbo*r algorithm, and counting standardized mean differences (SMD). Covariate balance after PSM were assessed using the R package *tableone* (version 0.13.2), employing the *χ*² test and *z*-test, with a significance level of α = 0.05 (two-tailed) in Table S2^45^. No significant differences were observed post-matching (SMD < 0.1, Figure S1).

The R package *qgraph* (version 1.9.8) was used to visualize the network of the matched data^46^. A regularized partial correlation network was constructed using the GGM and Least Absolute Shrinkage and Selection Operator (LASSO) techniques^41^. To ensure the selection of the most appropriate model, the Extended Bayesian Information Criterion (EBIC) was applied^47^. The network layout was arranged using the Fruchter-man-Reingold algorithm, which optimizes the placement of nodes to minimize edge overlap and enhance clarity. The AverageLayout function is used to compute the average position of the nodes in both groups, forming the same layout to facilitate visual comparison. At the same time, we only retained the edges with strong and significant correlations to more clearly identify the associations between symptoms.

Expected Influence (*EI*) is a centrality measure that considers the direct impact of a node on its neighbors, including both positive and negative relationships^26^. It was calculated by adding the edge weights connected to the node. Nodes with the highest *EI* values were often considered the most important^22,25^. The node strength centrality (*SC*) is the sum of the absolute values of all edges connected to the node. It reflects the strength of the between the symptom and other symptoms in the network and can also be used to determine the core symptoms in the network^26^. The core symptoms, identified by highest *EI* and *SC*, are pivotal as they can activate or sustain the psychopathological network. We computed the Bridge Expected Influence (*BEI*), which focuses on symptoms connecting different communities in the network^48^. *BEI* measures the impact of a node on other community nodes, highlighting its role as a bridge, and was calculated by adding the edge weights between node and nodes in different communities, providing insights into intercommunity interactions. The bridge symptom, identified by highest *BEI*. Predictability was defined as the variance of a node that could be explained by its adjacent nodes in the network model, which was estimated using the package *mgm* (version 1.2–14)^42^. In this network analysis, each mental health symptoms was represented as a node, and each pairwise association between symptoms was depicted as an edge. The thickness of the edges indicated the weight (*W*) of the regularized correlation coefficients, with thicker edges reflecting stronger associations. Green edges represented positive correlations, while red edges indicated negative correlations.

To compare the network invariance, global strength invariance, edge invariance and strength centrality invariance of different network groups, R package *Network Comparison Test* (version 2.2.2) was used to determine if there are statistical differences in global strength (the absolute sum of all edge weights) and local strength (the absolute sum of total edge weights on specific node) as well as network structure (the distributions of edge weights) among subgroups^24^. Global and local connectivity was compared by examining the differences in centrality for the same nodes across different networks, based on a bootstrapped subset (*n* = 1,000) from the stability test. Effect sizes were calculated to assess the magnitude of differences in node centrality^49^. To assess the accuracy and stability of the observed network models, R package *bootnet* (version 1.5.6) was employed to evaluate the accuracy and stability of each network model^50,51^. The 95% confidence intervals (*CI*) of the edge weights in the network were calculated using a non-parametric bootstrap test (*n* = 1,000), and an edge weight difference test was performed to prove the accuracy of the edge weight estimation. The Correlation Stability coefficient (CS-coefficient) was calculated using the case bootstrap test (*n* = 1,000). This metric assesses the stability of centrality indices in network models. The CS-coefficient evaluates how much of the sample can be dropped while maintaining a correlation of 0.7 with the original centrality indices. A value of at least 0.25 indicates adequate stability and a value above 0.5 indicates good stability^50^.

## Results

### Children characteristics

The demographic data according to their school stage are shown in Table 1.

**Table 1:** Participant demographics according to school stage.

Characteristics	Primary school	Junior high school	Total
Age (years, *Mean* ± *SD*)	9.93 ± 3.44	14.04 ± 2.49	11.24 ± 3.70
Sleep duration (hours, *Mean* ± *SD*)	9.16 ± 1.48	8.20 ± 1.70	8.86 ± 1.62
Gender			
Boys (n, %)	19,194 (51.6)	8,524 (49.3)	27,718 (50.9)
Girls (n, %)	17,989 (48.4)	8,781 (50.7)	26,770 (49.1)
Location			
Urban (n, %)	14,956 (40.2)	6,006 (34.7)	20,962 (38.5)
Town (n, %)	12,737 (34.3)	3,302 (19.1)	16,039 (29.4)
Village (n, %)	9,490 (25.5)	7,997 (46.2)	17,487 (32.1)
Only Child (n, %)	4,869 (13.1)	1,532 (8.9)	6,401 (11.7)
Sleep disorders (n, %)	2,496 (6.7)	4,158 (24.0)	6,654 (12.2)
Depression (n, %)	2,536 (6.8)	3,058 (17.7)	5,594 (10.3)
Anxiety (n, %)	2,739 (7.4)	3,300 (19.1)	6,039 (11.1)
Stress (n, %)	1,002 (2.7)	1,411 (8.2)	2,413 (4.4)
Participant Number	37,183	17,305	54,488

*SD* standard deviation. *Only Child* refers to children with no biological siblings, a demographic characteristic influenced by China’s one-child policy (1982–2016). This status may reflect unique family dynamics and resource allocation.

### Children mental health, life satisfaction and parental emotions

After PSM balancing the confounding factors between the two groups (Table S2), parental depression (12.1% vs. 12.4%, *x*^2^ = 0.01, *P >* 0.05), anxiety (15.2% vs. 15.8, *x*^2^ = 0.24, *P >* 0.05) and stress (6.4% vs. 5.9%, *x*^2^ = 1.96, *P >* 0.05) didn’t show significant difference in Table 2. The rate of depression in SDG children was significantly higher compared to NSDG (41.6% vs. 7.3%, *x*^2^ = 2,107.62, *P <* 0.01). The rate of anxiety in SDG children was significantly higher compared to NSDG (45.7% vs. 8.0%, *x*^2^ = 2,399.98, *P <* 0.01). The stress rate in SDG children was significantly higher than NSDG (23.4% vs. 2.6%, *x*^2^ = 1,267.84, *P <* 0.01). The proportion of children who were dissatisfied with their lives was higher in SDG (45.3% vs. 21.1%), while the proportion of children who were satisfied with their lives was higher in NSDG (65.9% vs. 38.4%) in Table 2.

**Table 2:** Comparison of baseline data before and after propensity score matching between the two groups.

Characteristics	Before matching				After matching			
	SDG (*n* = 6,654)	NSDG (*n* = 47,834)	x^2^/z	*P*	SDG (*n* = 6,635)	NSDG (*n* = 6,635)	x^2^/z	*P*
Age (years, *Mean* ± *SD*)	12.90 ± 3.80	11.00 ± 3.63	39.18	< 0.01	12.90 ± 3.46	12.90 ± 3.68	-0.77	0.44
Gender								
Male (n, %)	3,106 (46.7)	24,612 (51.5)	53.08	< 0.01	3,100 (46.7)	2,994 (45.1)	3.35	0.07
Female (n, %)	3,548 (53.3)	23,222 (48.5)			3,535 (53.3)	3,641 (54.9)		
School Stage								
Primary (n, %)	2,496 (37.5)	34,687 (72.5)	3,300.88	< 0.01	2,492 (37.6)	2,517 (37.9)	0.18	0.67
Junior High (n, %)	4,158 (62.5)	13,147 (27.5)			4,143 (62.4)	4,118 (62.1)		
Area								
Urban (n, %)	2,495 (37.5)	18,467 (38.6)	147.29	< 0.01	2,489 (37.5)	2,489 (37.5)	0	1
Town (n, %)	1,630 (24.5)	14,409 (30.1)			1,624 (24.5)	1,624 (24.5)		
Village (n, %)	2,529 (38.0)	14,958 (31.3)			2,522 (38.0)	2,522 (38.0)		
Only Child (n, %)	662 (9.9)	5,739 (12.0)	23.45	< 0.01	656 (9.9)	656 (9.9)	0	1
Parental depression (n, %)	809 (12.2)	5,844 (12.2)	0.14	0.71	806 (12.1)	825 (12.4)	0.01	0.94
Parental anxiety (n, %)	1,009 (15.2)	7,134 (14.9)	0.06	0.81	1,006 (15.2)	1,046 (15.8)	0.24	0.63
Parental stress (n, %)	423 (6.4)	2,943 (6.2)	0.20	0.65	422 (6.4)	390 (5.9)	1.96	0.16
Depression (n, %)	2,765 (41.6)	2,829 (5.9)	8,050.13	< 0.01	2,757 (41.6)	484 (7.3)	2,107.62	< 0.01
Anxiety (n, %)	3,038 (45.7)	3,001 (6.3)	9,189.64	< 0.01	3,030 (45.7)	529 (8.0)	2,399.98	< 0.01
Stress (n, %)	1,553 (23.3)	860 (1.8)	6,399.39	< 0.01	1,550 (23.4)	171 (2.6)	1,267.84	< 0.01
Life satisfaction								
Satisfied	2,554 (38.4)	33,781 (70.6)	3,066.26	< 0.01	2,549 (38.4)	4,371 (65.9)	1,091.56	< 0.01
Neutral	1,085 (16.3)	5,430 (11.4)			1,079 (16.3)	867 (13.0)		
Dissatisfied	3,015 (45.3)	8,623 (18.0)			3,007 (45.3)	1,397 (21.1)		
Sleep duration (hours, *Mean* ± SD)	7.43 ± 2.14	9.05 ± 1.42	-59.88	< 0.01	7.43 ± 2.14	8.80 ± 1.54	-42.3	< 0.01

NSDG non-sleep disorders group, SDG sleep disorders group, SD standard deviation, *x*^2^ was used to assess the difference between SDG and NSDG in proportions, *z* was employed to determine the statistical significance of the difference in means between two groups, *P* < 0.05 suggests there’s likely a significant difference between the two groups.

### Children mental health symptoms and association with parental emotions

**Fig. 1:**
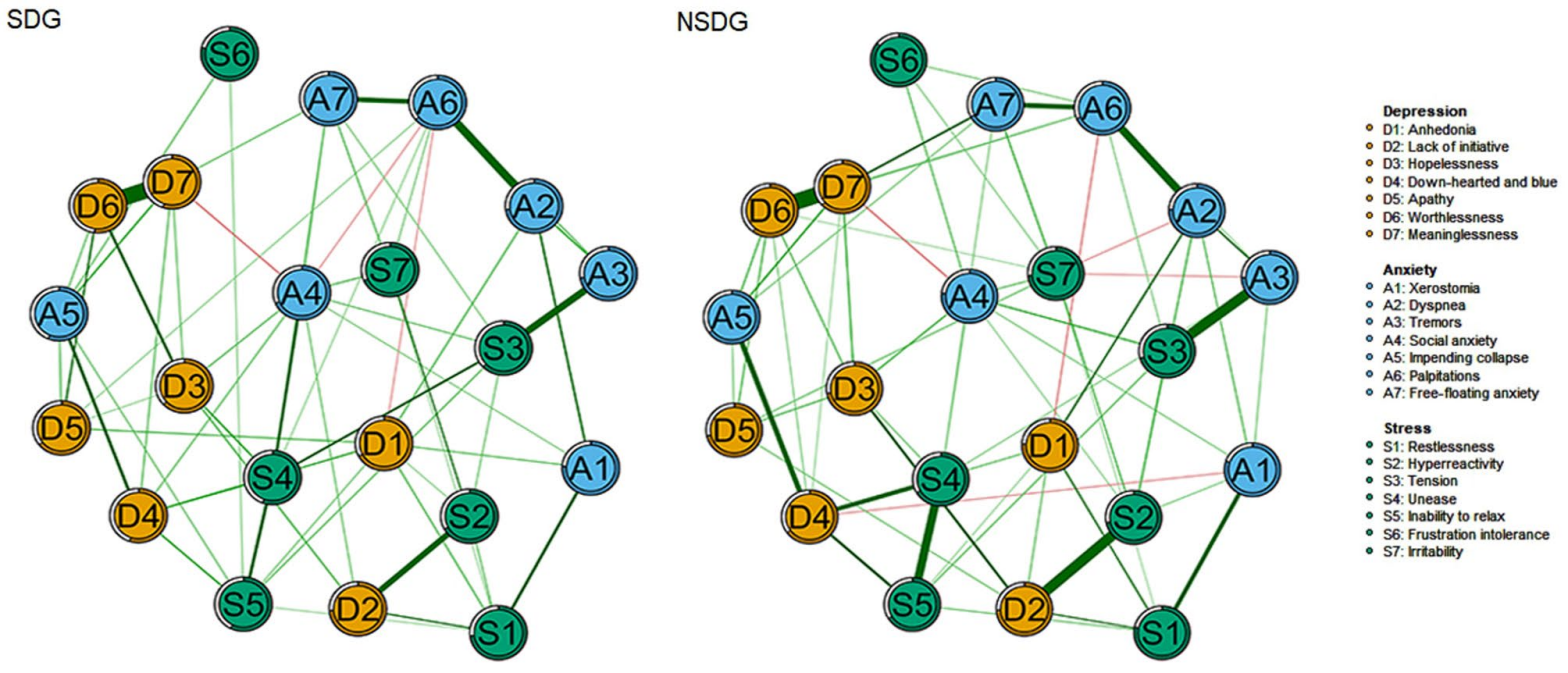
Mental health symptoms regularization partial correlations network between the two groups. SDG sleep disorders group, NSDG non-sleep disorders group.

The network structure of mental health symptoms in the two groups after matching is illustrated in Fig. 1, nodes represent symptoms, circles represent predictability, green edges represent positive associations, red edges represent negative associations between nodes, and the thickness of the edges indicated the weight of the correlations. In the SDG network, symptom predictability averaged 57%. The strongest edges involved “D7: *Meaninglessness*-D6: *Worthlessness*” (*W* = 0.52, Table S3) and “A6: *Palpitations-*A2: *Dyspnea*” (*W* = 0.33, Table S3). In the NSDG network, symptom predictability averaged 48.3%. The strongest edges involved “D7: *Meaninglessness*-D6: *Worthlessness*” (*W* = 0.41, Table S4) and “D2: *Lack of initiative*-S2: *Hyperreactivity”* (*W* = 0.33, Table S4).

**Fig. 2:**
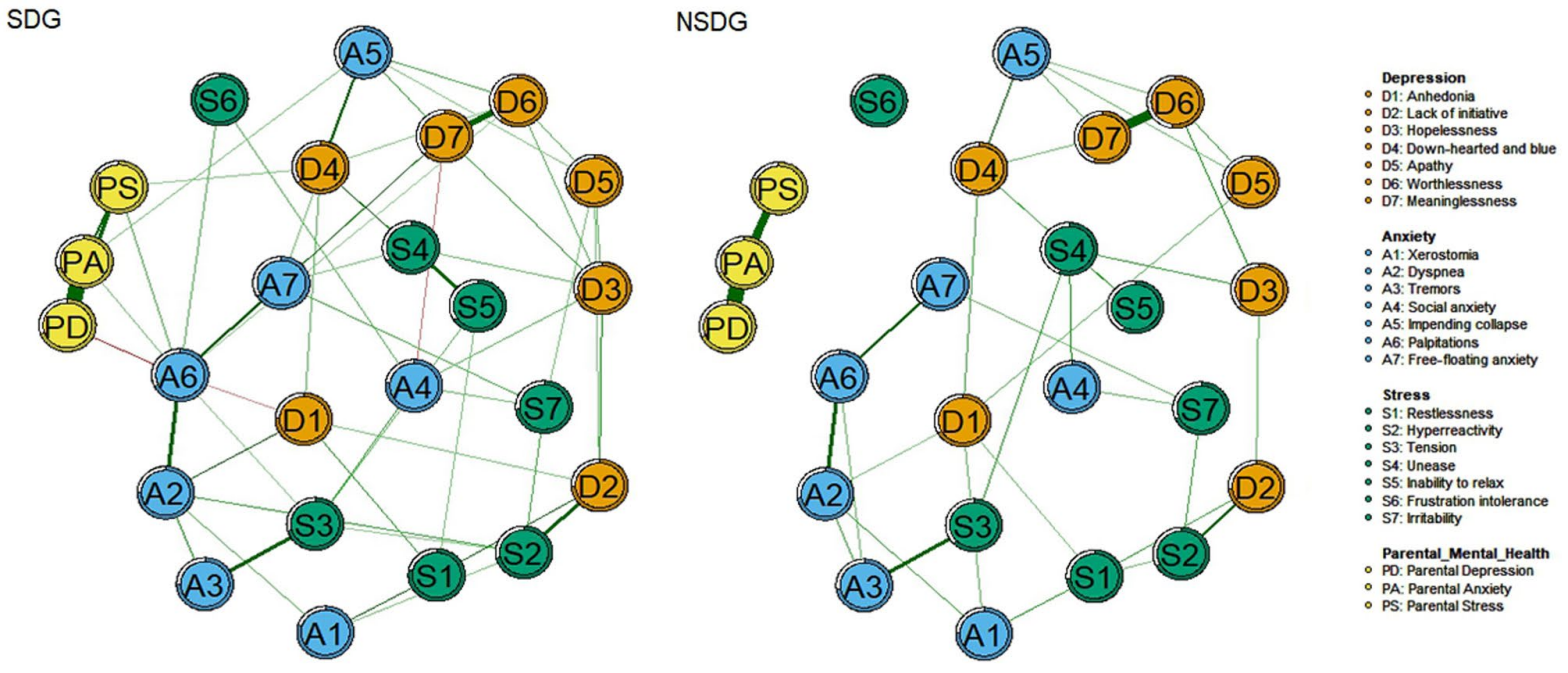
Mental health symptoms and parental emotions regularization partial correlations network between the two groups. SDG sleep disorders group, NSDG non-sleep disorders group.

There were no nodes in the NSDG showed any connections with PA: *Parental anxiety*, PD: *Parental depression* and PS: *Parental stress* in Fig. 2. However, in the SDG (Fig. 2), node A6: *Palpitations* exhibited connections to PD: *Parental depression* (*W*=-0.19, Table S5), PA: *Parental anxiet*y (*W* = 0.11, Table S5), and PS: *Parental stress* (*W* = 0.15, Table S5). Node D4: *Down-hearted and blue* and PS: *Parental stress* (*W* = 0.10, Table S5), node A5: *Impending collapse* and PA: *Parental anxiet*y (*W* = 0.11, Table S5) had connections.

**Fig. 3:**
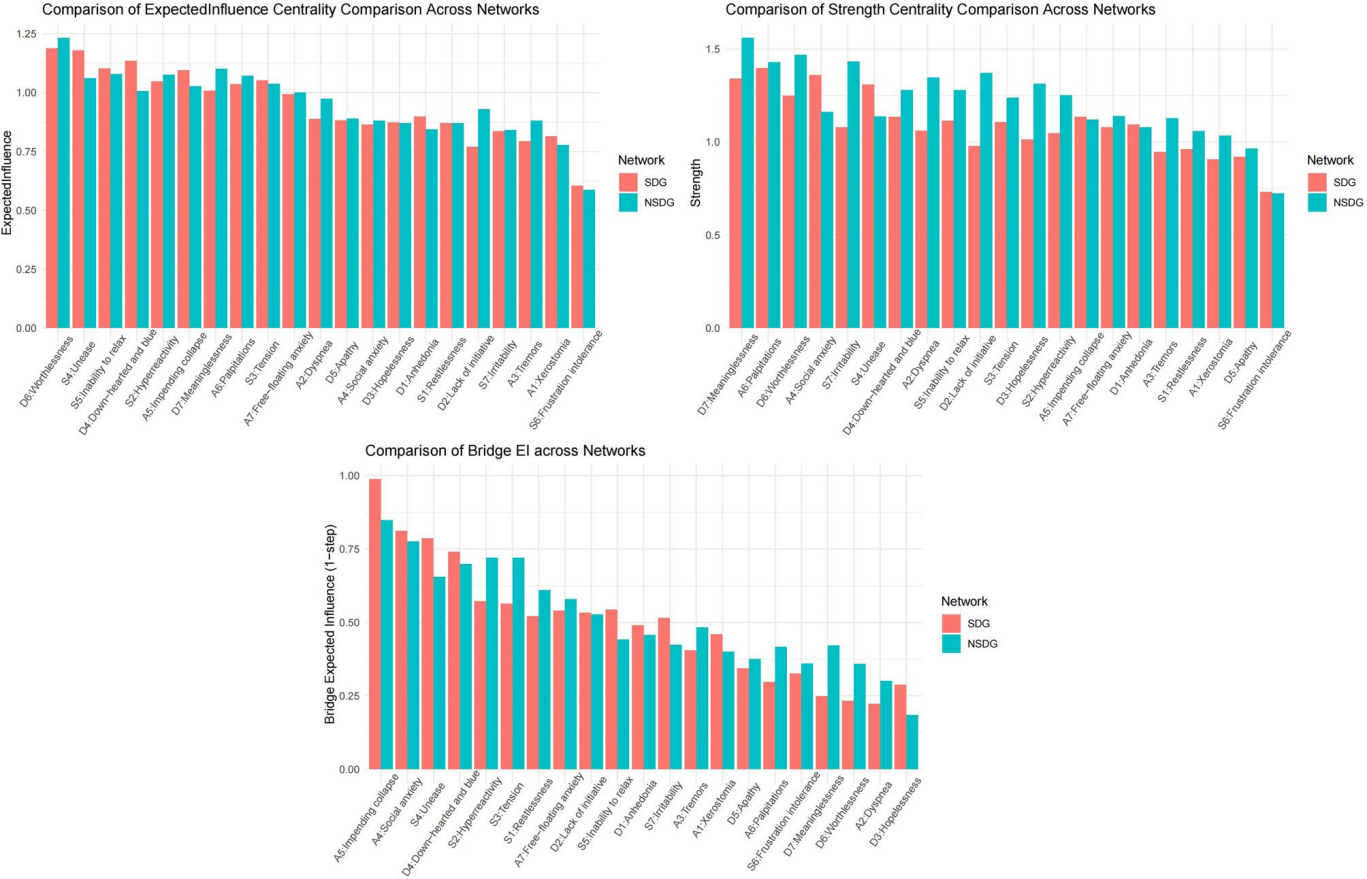
Node centrality of different networks. *SDG* sleep disorders group. *NSDG* non-sleep disorders group.

Table S7 and Fig. 3 showed the node centrality value by different groups. The node D6: *Worthlessness* (*EI* = 1.19) had the highest *EI* in SDG. The node A6: *Palpitations* (*SC* = 1.39) had the highest *SC* in SDG. Indicating the exacerbation of palpitations and worthlessness tended to precipitate a corresponding increase in the other symptoms. The node D6: *Worthlessness* (*EI* = 1.23) had the highest *EI*, and the node D7: *Meaninglessness* (*SC* = 1.56) had the highest *SC* in the NSDG. Indicating worthlessness and meaninglessness were most influential in the whole network model and could affect more symptoms. The node A5: *Impending collapse* (SDG: *BEI* = 0.99, NSDG: *BEI* = 0.85) had the highest *BEI* values in both groups. This suggested that the association between depression, anxiety, and stress was most likely mediated through this symptom. The node A5: *Impending collapse* and D4: *Down-hearted and blue* had strongest connection in SDG (*W* = 0.21, Table S3) and NSGD (*W* = 0.25, Table S4).

### Comparison of global and local connectivity of two networks

The network comparison test revealed significant differences in both network structure invariance (*M* = 0.15, *P* < 0.01) and global strength invariance (SDG: *S* = 11.48, NSDG: *S* = 12.76; *S*_*diff*_=1.28, *P* < 0.01). The edge weights of “D6: *Worthlessness*-D7: *Meaninglessness*” (SDG: *W* = 0.52, NSDG: *W* = 0.41), “A6: *Palpitations*-A2: *Dyspnea*” (SDG: *W* = 0.33, NSDG: *W* = 0.29), and “A6: *Palpitations*-A7: *Free-floating anxiety*” (SDG: *W* = 0.27, NSDG: *W* = 0.25), “A4: *Social anxiety*-S4: *Unease*” (SDG: *W* = 0.20, NSDG: *W* = 0.09) and “S4: *Unease*-S3: *Tension*” (SDG: *W* = 0.20, NSDG: *W* = 0.06) were larger in SDG (Tables S3 and S4). “A6: *Palpitations*-PD: *Parental depression*” (SDG: W = -0.19, NSDG: W = 0), “A6: *Palpitations*-PA: *Parental anxiety*” (SDG: W = 0.11, NSDG: W = 0), “A6: *Palpitations*-PS: *Parental stress*” (SDG: W = 0.15, NSDG: W = 0), “A5: *Impending collapse* - PA: *Parental anxiety*” (SDG: W = 0.11, NSDG: W = 0) and “D4: *Down-hearted and blue* - PS: *Parental stress*” (SDG: W = 0.10, NSDG: W = 0) were stronger in SDG (Table S5 and Table S6). These edges in SDG were nonzero in more than 90% of bootstraps.

**Fig. 4:**
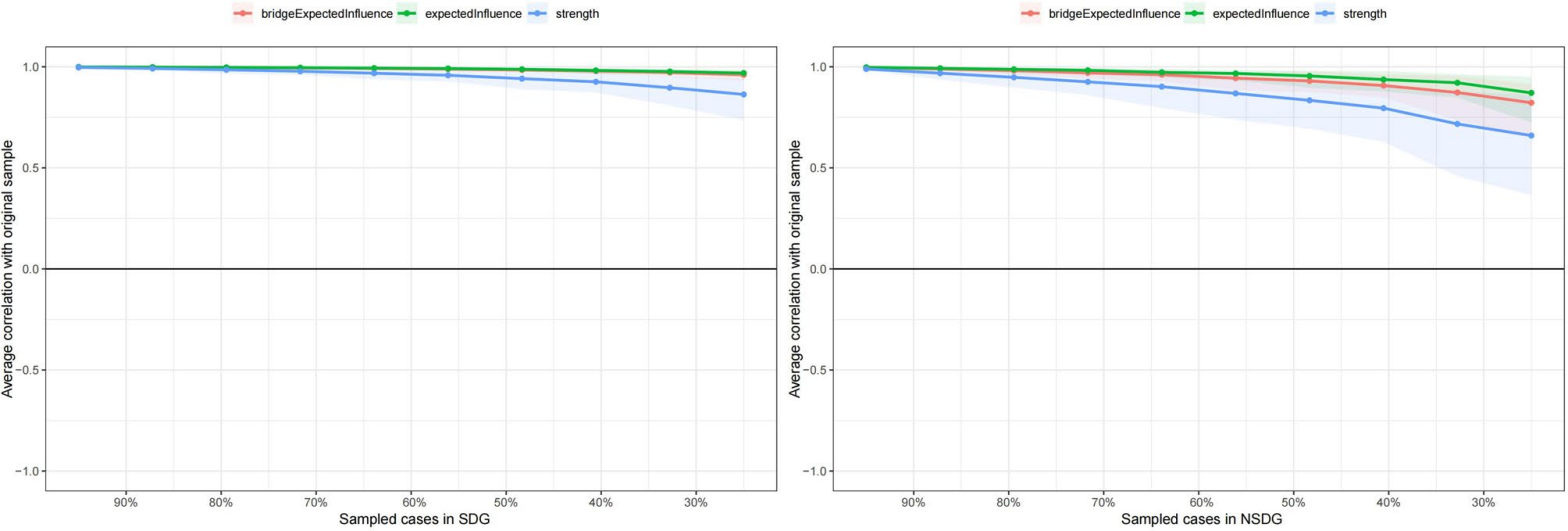
Average correlations between centrality indices of two networks sampled with persons dropped and the original sample.

Average correlations between centrality indices of two networks sampled with persons dropped and the original sample in Fig. 4. The correlation coefficients of SDG and NSDG node *EI*, *SC*, and *BEI* are all above 0.5, indicating that the centrality of the nodes is relatively stable and less sensitive to changes in sample size.

The comparison results of the bootstrapping test node average centrality are shown in Table S8, except for node S5: *Inability to relax*, node S1: *Restlessness*, node D5: *Apathy*, node D1: *Anhedonia*, node A7: *Free-floating anxiety* and node A5: *Impending collapse*, all other symptoms were statistically different between the two groups.

The edge weight stability CS-coefficient was both 0.75 and the 95% CI showed good accuracy in the network structure in Figures S2 and S3. Tests for differences in edges show that most non-zero edges in the network were statistically significant differences in Figures S4 and S5.

Figure S6 shows the network of mental health symptoms in the two groups before matching and multiple imputations, with a similar outcome. Subgroup analysis of gender, school stage, area, and whether the child is the only child (Figures S7-S10) found significant differences in network structure and strength between SDG and NSDG.

## Discussion

The current investigation revealed that the rates of SD in junior high school were consistent with previous epidemiological findings^5^. Higher proportion of female children in the SDG may reflect sex differences in sleep regulation during puberty, potentially linked to hormonal fluctuations affecting sleep architecture^2^. Non-only children from rural or township areas exhibited elevated risks, which could be associated with limited educational resources in underdeveloped regions and suboptimal sleep environments (e.g., noise pollution, overcrowded households)^52^. Additionally, older children and those in higher grades showed increased vulnerability to sleep quality deterioration^53^. Among children with SD, the rates of these mental health issues were significantly higher than those without. It confirms disturbed sleep can affect psychological problems in adolescents^1,2^. However, there was no difference in the incidence of mental health problems among different groups of parents, which is worth further exploration.

After balancing the above confounding factors, network analysis found palpitations (A6) were positively associated with parental anxiety and stress in SDG, although the incidence of depression, anxiety and stress among parents is relatively consistent. Network analysis found that mental health symptom network had higher average predictability in sleep-disordered children, higher predictability indicates stronger interdependence among adjacent symptoms. In NSDG, lower predictability suggests disrupted symptom networks, where interventions targeting central nodes may not have broader effects. The subjective feeling of *palpitations* (A6) is the core symptom of mental health in children with SD. It may reflect autonomic nervous system dysregulation and psychological hyperarousal, a bidirectional pathway^1^. In Chinese culture, academic performance and social reputation are highly valued in family, creating stressors that disrupt sleep and amplify social evaluation fears^5^. Sleep deprivation may further impair emotional regulation, exacerbating feelings of uneasiness. The prevalence of mental health problems and sleep disturbances increases with school stage, largely due to mounting academic pressure and parental stressors. Excessive focus on academic performance, combined with suppressed interests and hobbies, can aggravate sleep problems and contribute to mental health challenges^12^. These stressors are particularly problematic for children with SD, who tend to overthink, further concealing underlying psychological symptoms and complicating early intervention efforts.

Palpitations, the somatic subjective experience symptom awakened by anxiety, had the highest strength in SDG. This may be related to disturbances of the sympathetic nervous system, which can lead to irregular heart rhythms during SD^1^. Children’s naturally basal heart rate could also be affected by researchers’ and caregivers’ clinical assessments^11^. The strong edge weight between palpitations (A6) and dyspnea (A2) in the network of children with SD may show a kind of normal physiological phenomenon. Respiratory sinus arrhythmia (RSA) is common in children and usually does not require treatment^54^. However, sleep problems may contribute to the abnormality of this phenomenon. The central role of palpitations (A6) in children with sleep disorders (SDG) is a pivotal finding, reflecting both physiological and psychological mechanisms. Physiologically, sleep disorders disrupt the sympathetic-parasympathetic balance, leading to heightened sympathetic activation during wakefulness and insufficient parasympathetic regulation during rest^1,54^. This dysregulation manifests as subjective feelings of irregular heartbeat, which are strongly linked to anxiety arousal (i.e., edges with dyspnea and free-floating anxiety in Fig. 1). In children, whose cardiovascular systems are still developing, such somatic sensations may be particularly salient, serving as a bridge between poor sleep and emotional distress^54^. Psychologically, palpitations act as a somatic marker of unresolved anxiety, amplifying the feedback loop between sleep and mental health. Children often struggle to articulate abstract emotional states (e.g., “I feel anxious”) but can readily report physical symptoms like a “racing heart”^11^. This makes palpitations a detectable entry point for intervention—targeting this symptom may disrupt the network by reducing physiological arousal, which in turn alleviates anxiety and improves sleep quality. Cognitive-behavioral therapy (CBT) adapted for children could include biofeedback or mindfulness exercises to normalize heart rate variability, thereby breaking the cycle of hyperarousal^55^. Clinically, prioritizing palpitations in sleep-disordered children aligns with the biopsychosocial model, which emphasizes integrating somatic and psychological interventions^14^. Healthcare providers can screen for both sleep quality (via PSQI) and somatic anxiety symptoms (e.g., palpitations, dyspnea) in routine pediatric visits. School-based programs might incorporate stress management techniques (e.g., deep breathing exercises) to reduce sympathetic overactivity, particularly in children reporting recurrent heart-rate irregularities.

Worthlessness (D6) exhibited the highest Expected Influence (*EI*), and together with Meaninglessness (D7), demonstrated the strongest association within the mental health network structures of children. These symptoms are important clinical manifestations of depression^56^. Primary and secondary school students are in the stage of identity exploration, and a lack of opportunities to broaden their horizons may easily lead to a sense of meaninglessness in their lives. Symptoms such as social anxiety (A4), unease (S4), and tension (S3) are more pronounced in children with SD, who exhibit signs of social withdrawal and emotional distress. The weaker global strength of mental health symptoms may indicate an early warning signal for an impending depressive state change in children with SD. According to the network destabilization and transition (NDT) model, depressive states can become “stuck”, requiring external disturbances to disrupt the system^57^. Once the model is disrupted, the system may fluctuate between states until stabilizing in either a worse or better equilibrium. That may mask the expression of their underlying feeling and fail to resolve their issues. This tendency to internalize their struggles can prevent the early recognition and treatment of emotional difficulties, exacerbating both their sleep and psychological problems^20^. Primary and secondary school students are in a critical period of self-concept formation. Parental pressure and excessive focus on academic performance may suppress their interests and hobbies and thus aggravate sleep problems^58^. In this case, students may show a state of unconscious “resistance” action to learning in classes, that is, they are eager to change or grow in their hearts but are unable to actively participate in learning in actual behavior^59,60^.

Impending collapse (A5), as a bridge symptom, can not only aggravate the occurrence of depression, anxiety and stress comorbidity in children, but also serve as a mediator for negative emotions from parents anxiety (PA) to children. Impending collapse is related to increased parental anxiety and is most strongly associated with being down-hearted and blue, which in turn is associated with increased parental stress. Freud’s psychoanalytic theory posits that children develop defense mechanisms, such as unconscious repression and denial, to manage psychological disturbances stemming from familial and social conflicts^61^. These mechanisms, while initially adaptive, may evolve into maladaptive patterns when overused^62^. For instance, under chronic stress, students might unconsciously adopt addictive behaviors, such as reversing day-night routines, to seek immediate emotional relief. This disruption of circadian rhythms not only perpetuates escapism but also exacerbates cognitive impairments (e.g., attention deficits, memory decline) due to sleep deprivation. Over time, such cycles of avoidance and physiological dysregulation may worsen clinical outcomes, including heightened anxiety, depressive symptoms, and diminished academic functioning. Recent research highlights how dopamine signals in the brain can reinforce such behaviors, potentially leading to detrimental habits^63^. A crucial brain signal linked to long-term memory falters in rats when they are deprived of sleep, and more neuronal types exhibited synaptic loss after sleep deprivation than during sleep in normal sleep-wake cycles^64^. Those findings might also help to explain why overall network connectivity is weaker in children with SD.

Additionally, in the SDG network, stronger social anxiety (A4) may further prove the lack of interaction and support from elders and peers in children with SD^65^. Social phobia can intensify circadian disorders, depriving children of crucial psychological support^66^. Social anxiety (A4) was strongly associated with unease (S4) and interacted with the inability to relax (S5) and downhearted blue (D4) in a reinforcing feedback loop. SD may enhance their experience of social anxiety as well as their feelings of depression and restlessness. Moreover, social isolation deprives children of an important psychological support system, increasing their loneliness and helplessness when facing stress, thus exacerbating the severity of psychological problems. Parental factors, such as marital status and education level, also play a crucial role in adolescent mental health^12^. And in our study, girls made up 53.3% of the SDG, though no significant differences in symptom networks were observed between boys and girls. Previous research suggests that girls may be more vulnerable to SD and related mental health issues due to hormonal changes during puberty^11^. However, further research is needed to explore this potential link in greater detail.

Furthermore, Chinese children and adolescents sleep an average of 8.86 hours per night, with junior high school students averaging 8.20 hours. Both figures fall below the official recommended sleep duration^13^. With evidence suggesting similar neural mechanisms underlying sleep and mental health, maintaining a regular wake-up schedule can improve sleep quality and alleviate symptoms of depression^16^. Among children and adolescents, an average of 60 min/day of moderate-to-vigorous intensity aerobic physical activity (MVPA) across the week provides physical and mental well-being^67,68^. Children are in a period of rapid physiological and nervous system development. Individuals at this stage are very sensitive to external pressure. Early school start time, heavy coursework, and high expectations from parents can harm their physical and mental health^2^. These stressors not only exacerbate SD but can also lead to the pathologization of mental health, manifesting in externalizing problems such as impulsivity, rebelliousness, and aggression. To address these issues, medical institutions can explore the interests of young patients and increase interaction and companionship by incorporating cognitive behavioral therapy, drama, and art elements into the treatment process^55^. Schools should ease the burden of excessive homework and off-campus tutoring for students undergoing compulsory education, cultivating students’ correct interests and hobbies, creating a healthy learning environment, and increasing companionship and care for children and adolescents^58^. Families also play a key role in the child’s health. Maintaining a warm family environment such as often encouragement and good parental relationships contributes to the healthy development of children both physically and mentally. By improving the disease literacy and care skills of caregivers, students can be better supported in coping with academic pressure and mental health problems. Establishing a management system that involves the collaboration of families, schools, communities, and medical institutions is an effective way to promote the children’s health care. This system can provide all-around support to ensure that children’s psychological and physiological needs are met at all levels.

## Conclusion

Our findings showed that children with SD experience higher rates of depression, anxiety, stress, and life dissatisfaction. Worthlessness and palpitations emerged as the core symptom in their mental health network, and impending collapse emerged as the bridge symptom can aggravate the occurrence of depression, anxiety and stress comorbidity in children. Notably, parental depression, anxiety and stress may also be associated with these symptoms. These symptoms provide theoretical basis for the targeted psychological intervention of Chinese children with SD in the future.

### Limitations and future directions

This study has several limitations that warrant consideration. First, it relies on self-reported data from children and their parents, which may be subject to recall bias, social desirability effects, or misinterpretation. While trained investigators clarified questionnaire items, subjective reporting could still introduce measurement error. Second, the cross-sectional design precludes causal inference, as we cannot determine whether sleep disorders precede mental health symptoms or vice versa. Lastly, although we controlled for key demographics via propensity score matching, residual confounding may persist, and the generalizability of findings to non-Chinese populations or children with severe psychiatric conditions is uncertain. While our sample from the central part of China provides valuable insights into Chinese children’s mental health, findings may not fully generalize to coastal or highly urbanized regions like Shanghai or Shenzhen.

Future research should address these limitations through longitudinal designs, tracking sleep quality and mental health symptoms over time to establish temporal precedence and causal pathways. Incorporating objective measures (e.g., actigraphy, AECG) alongside self-reports would enhance data validity. Additionally, cross-cultural studies are needed to determine if the central role of palpitations and social anxiety in sleep-disordered children generalizes to other populations, considering variations in symptom presentation and healthcare access.

## Supplementary Information

Below is the link to the electronic supplementary material.

Supplementary Material 1

## Data Availability

Except for the data provided in the supplementary material, the datasets used and/or analyzed in the current study are available from the corresponding author on reasonable request.
