# Supplementary material for: Mental health symptoms in Chinese children with sleep disorders and association with parental emotions
Source: Sci Rep. 2025 Aug 26;15:31367. doi: 10.1038/s41598-025-14305-4 (PMC12381202; doi:10.1038/s41598-025-14305-4)
Supplement: Supplementary file 1 — Supplementary Material 1 [file 41598_2025_14305_MOESM1_ESM.docx]

| **Table S1 Participant demographics** | | | | |
| --- | --- | --- | --- | --- |
| **Characteristics** | **Urban** | **Town** | **Village** | **Total** |
| Total Children | 20,962 | 16,039 | 17,487 | 54,488 |
| Gender | | | | |
| Boys | 10,555(50.4%) | 8,293(51.7%) | 8,870(50.7%) | 27,718(50.9%) |
| Girls | 10,407(49.6%) | 7,746(48.3%) | 8,617(49.3%) | 26,770(49.1%) |
| School Stage | | | | |
| Primary | 14,956(71.3%) | 12,737(79.4%) | 9,490(54.3%) | 37,183(68.2%) |
| Junior High | 6,006(28.7%) | 3,302(20.6%) | 7,997(45.7%) | 17,305(31.8%) |
| Age | | | | |
| 6-12 | 12,814(61.13%) | 10,896(67.93%) | 8,129(46.49%) | 31,839(58.43%) |
| 13-18 | 8,148(38.87%) | 5,143(32.07%) | 9,358(53.51%) | 22,649(41.57%) |
| Height | 149.82±(16.52) | 147.34±(15.88) | 151.58±(17.17) | 149.66±(16.63) |
| Weight | 51.83±(25.53) | 50.32±(24.32) | 55.78±(26.36） | 52.66±(25.55) |
| Sleep Duration | 8.78±(1.52) | 8.96±(1.51) | 8.85±(1.80) | 8.86±(1.62) |
| Only Child | 3,876(18.5%) | 1,434(8.9%) | 1,091(6.2%) | 6,401(11.7%) |
| Sleep Disorder | 2,495(11.9%) | 1,630(10.2%) | 2,529(14.5%) | 6,654(12.2%) |
| Depression | 2,031(9.7%) | 1,430(8.8%) | 2,133(12.2%) | 5,594(10.3%) |
| Anxiety | 2,213(10.6%) | 1,534(9.6%) | 2,292(13.1%) | 6,039(11.1%) |
| Stress | 921(4.4%) | 598(3.7%) | 894(5.1%) | 2,413(4.4%) |

*Note.* SD=Standard Deviation; Height (centimeters, cm)±SD; Weight (kilogram, kg)±SD; Sleep duration (hours)±SD

| **Table S2 Partial Correlation Matrix Edge Weight in SDG** | | | | | | | | | | | | | | | | | | | | | |
| --- | --- | --- | --- | --- | --- | --- | --- | --- | --- | --- | --- | --- | --- | --- | --- | --- | --- | --- | --- | --- | --- |
|  | D1 | D2 | D3 | D4 | D5 | D6 | D7 | A1 | A2 | A3 | A4 | A5 | A6 | A7 | S1 | S2 | S3 | S4 | S5 | S6 | S7 |
| D1 | - | 0.03 | 0.05 | 0.17 | 0.14 | 0.03 | - | 0.12 | 0.13 | - | - | 0.04 | -0.08 | - | 0.13 | 0.07 | - | -0.02 | 0.09 | - | - |
| D2 | 0.03 | - | 0.14 | - | 0.07 | - | - | 0.04 | 0.02 | -0.03 | 0.11 | - | -0.01 | -0.02 | 0.18 | 0.28 | - | -0.01 | 0.01 | -0.04 | - |
| D3 | 0.05 | 0.14 | - | 0.01 | 0.07 | 0.20 | 0.11 | - | -0.01 | - | 0.11 | - | - | -0.03 | - | - | - | 0.18 | 0.07 | -0.02 | -0.01 |
| D4 | 0.17 | - | 0.01 | - | 0.05 | 0.04 | 0.12 | - | - | 0.02 | 0.11 | 0.21 | - | - | 0.01 | 0.01 | 0.01 | 0.13 | 0.18 | 0.02 | 0.03 |
| D5 | 0.14 | 0.07 | 0.07 | 0.05 | - | 0.18 | 0.02 | - | - | - | -0.02 | 0.12 | 0.07 | 0.03 | - | - | - | - | 0.04 | 0.04 | 0.05 |
| D6 | 0.03 | - | 0.20 | 0.04 | 0.18 | - | 0.51 | -0.01 | - | - | - | 0.11 | 0.05 | 0.03 | -0.02 | - | - | - | - | - | 0.06 |
| D7 | - | - | 0.11 | 0.12 | 0.02 | 0.51 | - | -0.03 | - | 0.04 | -0.11 | 0.17 | 0.06 | 0.11 | - | - | -0.03 | - | - | - | 0.04 |
| A1 | 0.12 | 0.04 | - | - | - | -0.01 | -0.03 | - | 0.19 | 0.05 | 0.08 | - | 0.04 | - | 0.21 | 0.07 | 0.02 | 0.03 | - | 0.02 | - |
| A2 | 0.13 | 0.02 | -0.01 | - | - | - | - | 0.19 | - | 0.17 | -0.04 | - | 0.33 | 0.02 | 0.02 | 0.01 | 0.06 | - | -0.01 | 0.03 | -0.03 |
| A3 | - | -0.03 | - | 0.02 | - | - | 0.04 | 0.05 | 0.17 | - | 0.01 | - | 0.12 | 0.03 | - | 0.06 | 0.30 | 0.05 | - | - | -0.04 |
| A4 | - | 0.11 | 0.11 | 0.11 | -0.02 | - | -0.11 | 0.08 | -0.04 | 0.01 | - | -0.02 | -0.07 | 0.08 | - | 0.04 | 0.11 | 0.20 | 0.05 | 0.07 | 0.13 |
| A5 | 0.04 | - | - | 0.21 | 0.12 | 0.11 | 0.17 | - | - | - | -0.02 | - | 0.06 | 0.06 | 0.01 | - | - | 0.03 | 0.10 | 0.11 | 0.07 |
| A6 | -0.08 | -0.01 | - | - | 0.07 | 0.05 | 0.06 | 0.04 | 0.33 | 0.12 | -0.07 | 0.06 | - | 0.26 | -0.02 | - | - | 0.07 | - | 0.05 | 0.09 |
| A7 | - | -0.02 | -0.03 | - | 0.03 | 0.03 | 0.11 | - | 0.02 | 0.03 | 0.08 | 0.06 | 0.26 | - | - | 0.03 | 0.09 | 0.12 | 0.01 | 0.05 | 0.13 |
| S1 | 0.13 | 0.18 | - | 0.01 | - | -0.02 | - | 0.21 | 0.02 | - | - | 0.01 | -0.02 | - | - | 0.12 | 0.05 | - | 0.07 | 0.04 | 0.07 |
| S2 | 0.07 | 0.28 | - | 0.01 | - | - | - | 0.07 | 0.01 | 0.06 | 0.04 | - | - | 0.03 | 0.12 | - | 0.10 | - | 0.03 | 0.04 | 0.18 |
| S3 | - | - | - | 0.01 | - | - | -0.03 | 0.02 | 0.06 | 0.30 | 0.11 | - | - | 0.09 | 0.05 | 0.10 | - | 0.20 | 0.11 | 0.03 | - |
| S4 | -0.02 | -0.01 | 0.18 | 0.13 | - | - | - | 0.03 | - | 0.05 | 0.20 | 0.03 | 0.07 | 0.12 | - | - | 0.20 | - | 0.21 | 0.02 | -0.04 |
| S5 | 0.09 | 0.01 | 0.07 | 0.18 | 0.04 | - | - | - | -0.01 | - | 0.05 | 0.10 | - | 0.01 | 0.07 | 0.03 | 0.11 | 0.21 | - | 0.09 | 0.04 |
| S6 | - | -0.04 | -0.02 | 0.02 | 0.04 | - | - | 0.02 | 0.03 | - | 0.07 | 0.11 | 0.05 | 0.05 | 0.04 | 0.04 | 0.03 | 0.02 | 0.09 | - | 0.06 |
| S7 | - | - | -0.01 | 0.03 | 0.05 | 0.06 | 0.04 | - | -0.03 | -0.04 | 0.13 | 0.07 | 0.09 | 0.13 | 0.07 | 0.18 | - | -0.04 | 0.04 | 0.06 | - |

| **Table S3 Partial Correlation Matrix Edge Weight in NSDG controls** | | | | | | | | | | | | | | | | | | | | | |
| --- | --- | --- | --- | --- | --- | --- | --- | --- | --- | --- | --- | --- | --- | --- | --- | --- | --- | --- | --- | --- | --- |
|  | D1 | D2 | D3 | D4 | D5 | D6 | D7 | A1 | A2 | A3 | A4 | A5 | A6 | A7 | S1 | S2 | S3 | S4 | S5 | S6 | S7 |
| D1 | - | 0.13 | 0.01 | 0.13 | 0.06 | 0.07 | - | 0.03 | 0.20 | -0.01 | - | 0.02 | -0.11 | 0.01 | 0.20 | - | - | 0.03 | 0.07 | - | - |
| D2 | 0.13 | - | 0.21 | - | 0.10 | - | -0.04 | - | -0.03 | -0.02 | 0.05 | -0.05 | -0.05 | - | 0.20 | 0.33 | - | - | 0.06 | -0.03 | 0.07 |
| D3 | 0.01 | 0.21 | - | - | 0.14 | 0.14 | 0.18 | - | -0.01 | 0.05 | 0.15 | 0.03 | 0.01 | -0.07 | -0.03 | 0.03 | -0.02 | 0.11 | 0.04 | -0.02 | -0.07 |
| D4 | 0.13 | - | - | - | - | 0.10 | 0.08 | -0.09 | - | -0.01 | 0.03 | 0.25 | 0.01 | - | 0.02 | -0.02 | - | 0.24 | 0.22 | -0.01 | 0.06 |
| D5 | 0.06 | 0.10 | 0.14 | - | - | 0.15 | 0.05 | - | 0.01 | - | - | 0.10 | 0.02 | 0.05 | - | 0.01 | - | -0.04 | 0.05 | 0.06 | 0.11 |
| D6 | 0.07 | - | 0.14 | 0.10 | 0.15 | - | 0.41 | 0.03 | - | 0.05 | 0.05 | 0.15 | 0.10 | - | -0.02 | -0.04 | -0.06 | - | - | 0.02 | 0.07 |
| D7 | - | -0.04 | 0.18 | 0.08 | 0.05 | 0.41 | - | 0.04 | - | 0.06 | -0.12 | 0.20 | 0.11 | 0.21 | -0.05 | - | - | - | -0.02 | - | - |
| A1 | 0.03 | - | - | -0.09 | - | 0.03 | 0.04 | - | 0.14 | 0.11 | 0.09 | 0.01 | - | 0.03 | 0.24 | 0.09 | 0.02 | 0.03 | - | 0.05 | -0.04 |
| A2 | 0.20 | -0.03 | -0.01 | - | 0.01 | - | - | 0.14 | - | 0.20 | - | 0.03 | 0.29 | 0.01 | 0.05 | 0.16 | 0.07 | - | -0.06 | - | -0.08 |
| A3 | -0.01 | -0.02 | 0.05 | -0.01 | - | 0.05 | 0.06 | 0.11 | 0.20 | - | - | - | 0.09 | - | - | 0.01 | 0.33 | 0.09 | - | 0.02 | -0.08 |
| A4 | - | 0.05 | 0.15 | 0.03 | - | 0.05 | -0.12 | 0.09 | - | - | - | - | -0.02 | 0.03 | - | 0.08 | 0.18 | 0.09 | - | 0.14 | 0.13 |
| A5 | 0.02 | -0.05 | 0.03 | 0.25 | 0.10 | 0.15 | 0.20 | 0.01 | 0.03 | - | - | - | 0.05 | 0.10 | - | - | 0.04 | - | 0.03 | 0.04 | 0.02 |
| A6 | -0.11 | -0.05 | 0.01 | 0.01 | 0.02 | 0.10 | 0.11 | - | 0.29 | 0.09 | -0.02 | 0.05 | - | 0.25 | - | 0.01 | 0.10 | - | 0.04 | 0.10 | 0.06 |
| A7 | 0.01 | - | -0.07 | - | 0.05 | - | 0.21 | 0.03 | 0.01 | - | 0.03 | 0.10 | 0.25 | - | - | 0.06 | 0.06 | 0.10 | - | - | 0.17 |
| S1 | 0.20 | 0.20 | -0.03 | 0.02 | - | -0.02 | -0.05 | 0.24 | 0.05 | - | - | - | - | - | - | 0.06 | 0.01 | - | 0.12 | - | 0.07 |
| S2 | - | 0.33 | 0.03 | -0.02 | 0.01 | -0.04 | - | 0.09 | 0.16 | 0.01 | 0.08 | - | 0.01 | 0.06 | 0.06 | - | 0.12 | - | -0.03 | 0.05 | 0.16 |
| S3 | - | - | -0.02 | - | - | -0.06 | - | 0.02 | 0.07 | 0.33 | 0.18 | 0.04 | 0.10 | 0.06 | 0.01 | 0.12 | - | 0.06 | 0.12 | 0.03 | -0.02 |
| S4 | 0.03 | - | 0.11 | 0.24 | -0.04 | - | - | 0.03 | - | 0.09 | 0.09 | - | - | 0.10 | - | - | 0.06 | - | 0.29 | - | 0.05 |
| S5 | 0.07 | 0.06 | 0.04 | 0.22 | 0.05 | - | -0.02 | - | -0.06 | - | - | 0.03 | 0.04 | - | 0.12 | -0.03 | 0.12 | 0.29 | - | 0.06 | 0.07 |
| S6 | - | -0.03 | -0.02 | -0.01 | 0.06 | 0.02 | - | 0.05 | - | 0.02 | 0.14 | 0.04 | 0.10 | - | - | 0.05 | 0.03 | - | 0.06 | - | 0.09 |
| S7 | - | 0.07 | -0.07 | 0.06 | 0.11 | 0.07 | - | -0.04 | -0.08 | -0.08 | 0.13 | 0.02 | 0.06 | 0.17 | 0.07 | 0.16 | -0.02 | 0.05 | 0.07 | 0.09 | - |

| **Table S4 Basic Centrality Information of Symptoms** | | | | | | | | |
| --- | --- | --- | --- | --- | --- | --- | --- | --- |
| **Symptoms** | **SDG** | | | | **NSDG controls** | | | |
|  | **Strength** | **Predictability** | **Bridge EI** | **Closeness** | **Strength** | **Predictability** | **Bridge EI** | **Closeness** |
| D1: *Anhedonia* | 1.09 | 0.67 | 0.49 | 0.39×10-2 | 1.08 | 0.42 | 0.46 | 0.41×10^-2^ |
| D2: *Lack of initiative* | 0.98 | 0.74 | 0.53 | 0.37×10-2 | 1.37 | 0.45 | 0.53 | 0.45×10^-2^ |
| D3: *Hopelessness* | 1.01 | 0.63 | 0.29 | 0.39×10-2 | 1.31 | 0.49 | 0.18 | 0.47×10^-2^ |
| D4: *Depression* | 1.14 | 0.55 | 0.74 | 0.40×10-2 | 1.28 | 0.58 | 0.70 | 0.40×10^-2^ |
| D5: *Apathy* | 0.92 | 0.64 | 0.34 | 0.34×10-2 | 0.96 | 0.49 | 0.37 | 0.35×10^-2^ |
| D6: *Worthlessness* | 1.25 | 0.52 | 0.23 | 0.39×10-2 | 1.47 | 0.63 | 0.36 | 0.41×10^-2^ |
| D7: *Meaninglessness* | 1.34 | 0.55 | 0.25 | 0.38×10-2 | 1.56 | 0.60 | 0.42 | 0.44×10^-2^ |
| A1: *Xerostomia* | 0.91 | 0.76 | 0.46 | 0.34×10-2 | 1.03 | 0.35 | 0.40 | 0.39×10^-2^ |
| A2: *Dyspnea* | 1.06 | 0.73 | 0.22 | 0.37×10-2 | 1.35 | 0.43 | 0.30 | 0.45×10^-2^ |
| A3: *Tremors* | 0.95 | 0.73 | 0.40 | 0.35×10-2 | 1.13 | 0.44 | 0.48 | 0.40×10^-2^ |
| A4: *Social anxiety* | 1.36 | 0.69 | 0.81 | 0.42×10-2 | 1.16 | 0.41 | 0.78 | 0.43×10^-2^ |
| A5: *Impending collapse*: | 1.14 | 0.56 | 0.99 | 0.37×10-2 | 1.12 | 0.58 | 0.85 | 0.39×10^-2^ |
| A6: *Palpitation* | 1.40 | 0.64 | 0.30 | 0.38×10-2 | 1.43 | 0.53 | 0.42 | 0.44×10^-2^ |
| A7: *Free-floating anxiety* | 1.08 | 0.62 | 0.54 | 0.39×10-2 | 1.14 | 0.55 | 0.58 | 0.44×10^-2^ |
| S1: *Restlessness* | 0.96 | 0.73 | 0.52 | 0.35×10-2 | 1.06 | 0.39 | 0.61 | 0.40×10^-2^ |
| S2: *Hyperreactivity* | 1.05 | 0.67 | 0.57 | 0.35×10-2 | 1.25 | 0.49 | 0.72 | 0.42×10^-2^ |
| S3: *Tension* | 1.11 | 0.63 | 0.56 | 0.38×10-2 | 1.24 | 0.50 | 0.72 | 0.42×10^-2^ |
| S4: *Unease* | 1.31 | 0.56 | 0.79 | 0.44×10-2 | 1.14 | 0.56 | 0.65 | 0.41×10^-2^ |
| S5: *Inability to relax* | 1.11 | 0.58 | 0.54 | 0.38×10-2 | 1.28 | 0.56 | 0.44 | 0.40×10^-2^ |
| S6: *Frustration intolerance* | 0.73 | 0.79 | 0.33 | 0.27×10-2 | 0.72 | 0.27 | 0.36 | 0.31×10^-2^ |
| S7: *Irritability* | 1.08 | 0.68 | 0.52 | 0.35×10-2 | 1.43 | 0.46 | 0.42 | 0.41×10^-2^ |

| **Table S5 Comparison of 1000 bootstrapping network centrality across groups.** | | | | |
| --- | --- | --- | --- | --- |
| **Symptom** | **M±SD** | | ***P*** | **Cohen’s d** |
|  | **SDG** | **NSDG** |  |  |
| S7: *Irritability* | 0.92±0.04 | 1.04±0.07 | <0.01 | -2.98 |
| S6: *Frustration intolerance* | 0.68±0.03 | 0.63±0.03 | <0.01 | 2.36 |
| S5: *Inability to relax* | 1.12±0.02 | 1.14±0.05 | <0.01 | -0.74 |
| S4: *Unease* | 1.17±0.03 | 1.04±0.04 | <0.01 | 5.20 |
| S3: *Tension* | 1.06±0.03 | 1.04±0.05 | 0.04 | 0.69 |
| S2: *Hyperreactivity* | 1.02±0.02 | 1.09±0.05 | <0.01 | -2.60 |
| S1: *Restlessness* | 0.87±0.03 | 0.87±0.05 | 0.21 | 0.00 |
| D7: *Meaninglessness* | 1.12±0.04 | 1.16±0.08 | 0.03 | -0.89 |
| D6: *Worthlessness* | 1.16±0.03 | 1.20±0.06 | <0.01 | -1.19 |
| D5: *Apathy* | 0.91±0.03 | 0.94±0.03 | 0.30 | -1.41 |
| D4: *Down-hearted and blue* | 1.14±0.01 | 1.07±0.05 | ＜0.01 | 2.75 |
| D3: *Hopelessness* | 0.92±0.03 | 0.98±0.07 | ＜0.01 | -1.58 |
| D2: *Lack of initiative* | 0.88±0.05 | 1.06±0.06 | ＜0.01 | -4.61 |
| D1: *Anhedonia* | 1.00±0.04 | 0.95±0.05 | 0.87 | 1.56 |
| A7: *Free-floating anxiety* | 1.01±0.03 | 1.04±0.05 | 0.44 | -1.03 |
| A6: *Palpitations* | 1.15±0.06 | 1.10±0.07 | 0.02 | 1.08 |
| A5: *Impending collapse* | 1.11±0.03 | 1.03±0.04 | 0.83 | 3.20 |
| A4: *Social anxiety* | 1.11±0.06 | 1.00±0.06 | 0.01 | 2.59 |
| A3: *Tremors* | 0.82±0.04 | 0.94±0.06 | 0.04 | -3.33 |
| A2: *Dyspnea* | 0.87±0.04 | 0.97±0.08 | ＜0.01 | -2.24 |
| A1: *Xerostomia* | 0.84±0.03 | 0.82±0.04 | 0.02 | 0.80 |

SDG sleep disorders group, NSDG Non-sleep disorders Group, M±SD the mean and standard deviation of centrality are derived from 1000 bootstrapping networks, Cohen’s d the positive/negative effect size indicates that the network node centrality of the SD group is higher/lower than NSDG, P < 0.05 suggests there’s likely a significant difference between the two groups.

| **Table S6 Partial Correlation Matrix Edge Weight in SDG controls after adding parental mental health** | | | | | | | | | | | | | | | | | | | | | | | | |
| --- | --- | --- | --- | --- | --- | --- | --- | --- | --- | --- | --- | --- | --- | --- | --- | --- | --- | --- | --- | --- | --- | --- | --- | --- |
|  | D1 | D2 | D3 | D4 | D5 | D6 | D7 | A1 | A2 | A3 | A4 | A5 | A6 | A7 | S1 | S2 | S3 | S4 | S5 | S6 | S7 | PD | PA | PS |
| D1 | - | 0.12 | - | 0.13 | - | - | - | - | 0.21 | - | - | - | -0.10 | - | 0.18 | - | - | - | - | - | - | - | - | - |
| D2 | 0.12 | - | 0.20 | - | 0.11 | - | - | - | - | - | - | - | - | - | 0.22 | 0.32 | - | - | - | - | - | - | - | - |
| D3 | - | 0.20 | - | - | 0.14 | 0.14 | 0.19 | - | - | - | 0.14 | - | - | - | - | - | - | 0.13 | - | - | - | - | - | - |
| D4 | 0.13 | - | - | - | - | 0.11 | - | - | - | - | - | 0.27 | - | - | - | - | - | 0.25 | 0.19 | - | - | - | - | 0.10 |
| D5 | - | 0.11 | 0.14 | - | - | 0.15 | - | - | - | - | - | 0.11 | - | - | - | - | - | - | - | - | 0.11 | - | - | - |
| D6 | - | - | 0.14 | 0.11 | 0.15 | - | 0.39 | - | - | - | - | 0.16 | 0.10 | - | - | - | - | - | - | - | - | - | - | - |
| D7 | - | - | 0.19 | - | - | 0.39 | - | - | - | - | -0.12 | 0.18 | 0.11 | 0.22 | - | - | - | - | - | - | - | - | - | - |
| A1 | - | - | - | - | - | - | - | - | 0.15 | - | - | - | - | - | 0.23 | 0.12 | - | - | - | - | - | - | - | - |
| A2 | 0.21 | - | - | - | - | - | - | 0.15 | - | 0.19 | - | - | 0.30 | - | - | 0.18 | - | - | - | - | - | - | - | - |
| A3 | - | - | - | - | - | - | - | - | 0.19 | - | - | - | - | - | - | - | 0.34 | - | - | - | - | - | - | - |
| A4 | - | - | 0.14 | - | - | - | -0.12 | - | - | - | - | - | - | - | - | - | 0.17 | - | - | 0.14 | 0.13 | - | - | - |
| A5 | - | - | - | 0.27 | 0.11 | 0.16 | 0.18 | - | - | - | - | - | - | 0.11 | - | - | - | - | - | - | - | - | 0.11 | - |
| A6 | -0.10 | - | - | - | - | 0.10 | 0.11 | - | 0.30 | - | - | - | - | 0.25 | - | - | 0.10 | - | - | 0.14 | - | -0.19 | 0.11 | 0.15 |
| A7 | - | - | - | - | - | - | 0.22 | - | - | - | - | 0.11 | 0.25 | - | - | - | - | 0.11 | - | - | 0.16 | - | - | - |
| S1 | 0.18 | 0.22 | - | - | - | - | - | 0.23 | - | - | - | - | - | - | - | - | - | - | 0.11 | - | - | - | - | - |
| S2 | - | 0.32 | - | - | - | - | - | 0.12 | 0.18 | - | - | - | - | - | - | - | 0.10 | - | - | - | 0.16 | - | - | - |
| S3 | - | - | - | - | - | - | - | - | - | 0.34 | 0.17 | - | 0.10 | - | - | 0.10 | - | - | 0.13 | - | - | - | - | - |
| S4 | - | - | 0.13 | 0.25 | - | - | - | - | - | - | - | - | - | 0.11 | - | - | - | - | 0.30 | - | - | - | - | - |
| S5 | - | - | - | 0.19 | - | - | - | - | - | - | - | - | - | - | 0.11 | - | 0.13 | 0.30 | - | - | - | - | - | - |
| S6 | - | - | - | - | - | - | - | - | - | - | 0.14 | - | 0.14 | - | - | - | - | - | - | - | - | - | - | - |
| S7 | - | - | - | - | 0.11 | - | - | - | - | - | 0.13 | - | - | 0.16 | - | 0.16 | - | - | - | - | - | - | - | - |
| PD | - | - | - | - | - | - | - | - | - | - | - | - | -0.19 | - | - | - | - | - | - | - | - | - | 0.74 | 0.31 |
| PA | - | - | - | - | - | - | - | - | - | - | - | 0.11 | 0.11 | - | - | - | - | - | - | - | - | 0.74 | - | 0.29 |
| PS | - | - | - | 0.10 | - | - | - | - | - | - | - | - | 0.15 | - | - | - | - | - | - | - | - | 0.31 | 0.29 | - |

| **Table S7 Partial Correlation Matrix Edge Weight in NSDG controls after adding parental mental health** | | | | | | | | | | | | | | | | | | | | | | | | |
| --- | --- | --- | --- | --- | --- | --- | --- | --- | --- | --- | --- | --- | --- | --- | --- | --- | --- | --- | --- | --- | --- | --- | --- | --- |
|  | D1 | D2 | D3 | D4 | D5 | D6 | D7 | A1 | A2 | A3 | A4 | A5 | A6 | A7 | S1 | S2 | S3 | S4 | S5 | S6 | S7 | PD | PA | PS |
| D1 | - | - | - | 0.16 | 0.14 | - | - | 0.13 | 0.12 | - | - | - | - | - | 0.12 | - | - | - | - | - | - | - | - | - |
| D2 | - | - | 0.14 | - | - | - | - | - | - | - | - | - | - | - | 0.18 | 0.28 | - | - | - | - | - | - | - | - |
| D3 | - | 0.14 | - | - | - | 0.21 | - | - | - | - | - | - | - | - | - | - | - | 0.18 | - | - | - | - | - | - |
| D4 | 0.16 | - | - | - | - | - | 0.13 | - | - | - | - | 0.23 | - | - | - | - | - | 0.14 | 0.17 | - | - | - | - | - |
| D5 | 0.14 | - | - | - | - | 0.18 | - | - | - | - | - | 0.13 | - | - | - | - | - | - | - | - | - | - | - | - |
| D6 | - | - | 0.21 | - | 0.18 | - | 0.51 | - | - | - | - | 0.12 | - | - | - | - | - | - | - | - | - | - | - | - |
| D7 | - | - | - | 0.13 | - | 0.51 | - | - | - | - | - | 0.16 | - | - | - | - | - | - | - | - | - | - | - | - |
| A1 | 0.13 | - | - | - | - | - | - | - | 0.18 | - | - | - | - | - | 0.20 | - | - | - | - | - | - | - | - | - |
| A2 | 0.12 | - | - | - | - | - | - | 0.18 | - | 0.17 | - | - | 0.32 | - | - | - | - | - | - | - | - | - | - | - |
| A3 | - | - | - | - | - | - | - | - | 0.17 | - | - | - | 0.13 | - | - | - | 0.30 | - | - | - | - | - | - | - |
| A4 | - | - | - | - | - | - | - | - | - | - | - | - | - | - | - | - | - | 0.21 | - | - | 0.13 | - | - | - |
| A5 | - | - | - | 0.23 | 0.13 | 0.12 | 0.16 | - | - | - | - | - | - | - | - | - | - | - | - | - | - | - | - | - |
| A6 | - | - | - | - | - | - | - | - | 0.32 | 0.13 | - | - | - | 0.26 | - | - | - | - | - | - | - | - | - | - |
| A7 | - | - | - | - | - | - | - | - | - | - | - | - | 0.26 | - | - | - | - | - | - | - | 0.13 | - | - | - |
| S1 | 0.12 | 0.18 | - | - | - | - | - | 0.20 | - | - | - | - | - | - | - | 0.12 | - | - | - | - | - | - | - | - |
| S2 | - | 0.28 | - | - | - | - | - | - | - | - | - | - | - | - | 0.12 | - | - | - | - | - | 0.18 | - | - | - |
| S3 | - | - | - | - | - | - | - | - | - | 0.30 | - | - | - | - | - | - | - | 0.18 | - | - | - | - | - | - |
| S4 | - | - | 0.18 | 0.14 | - | - | - | - | - | - | 0.21 | - | - | - | - | - | 0.18 | - | 0.21 | - | - | - | - | - |
| S5 | - | - | - | 0.17 | - | - | - | - | - | - | - | - | - | - | - | - | - | 0.21 | - | - | - | - | - | - |
| S6 | - | - | - | - | - | - | - | - | - | - | - | - | - | - | - | - | - | - | - | - | - | - | - | - |
| S7 | - | - | - | - | - | - | - | - | - | - | 0.13 | - | - | 0.13 | - | 0.18 | - | - | - | - | - | - | - | - |
| PD | - | - | - | - | - | - | - | - | - | - | - | - | - | - | - | - | - | - | - | - | - | - | 0.69 | 0.22 |
| PA | - | - | - | - | - | - | - | - | - | - | - | - | - | - | - | - | - | - | - | - | - | 0.69 | - | 0.45 |
| PS | - | - | - | - | - | - | - | - | - | - | - | - | - | - | - | - | - | - | - | - | - | 0.22 | 0.45 | - |

**
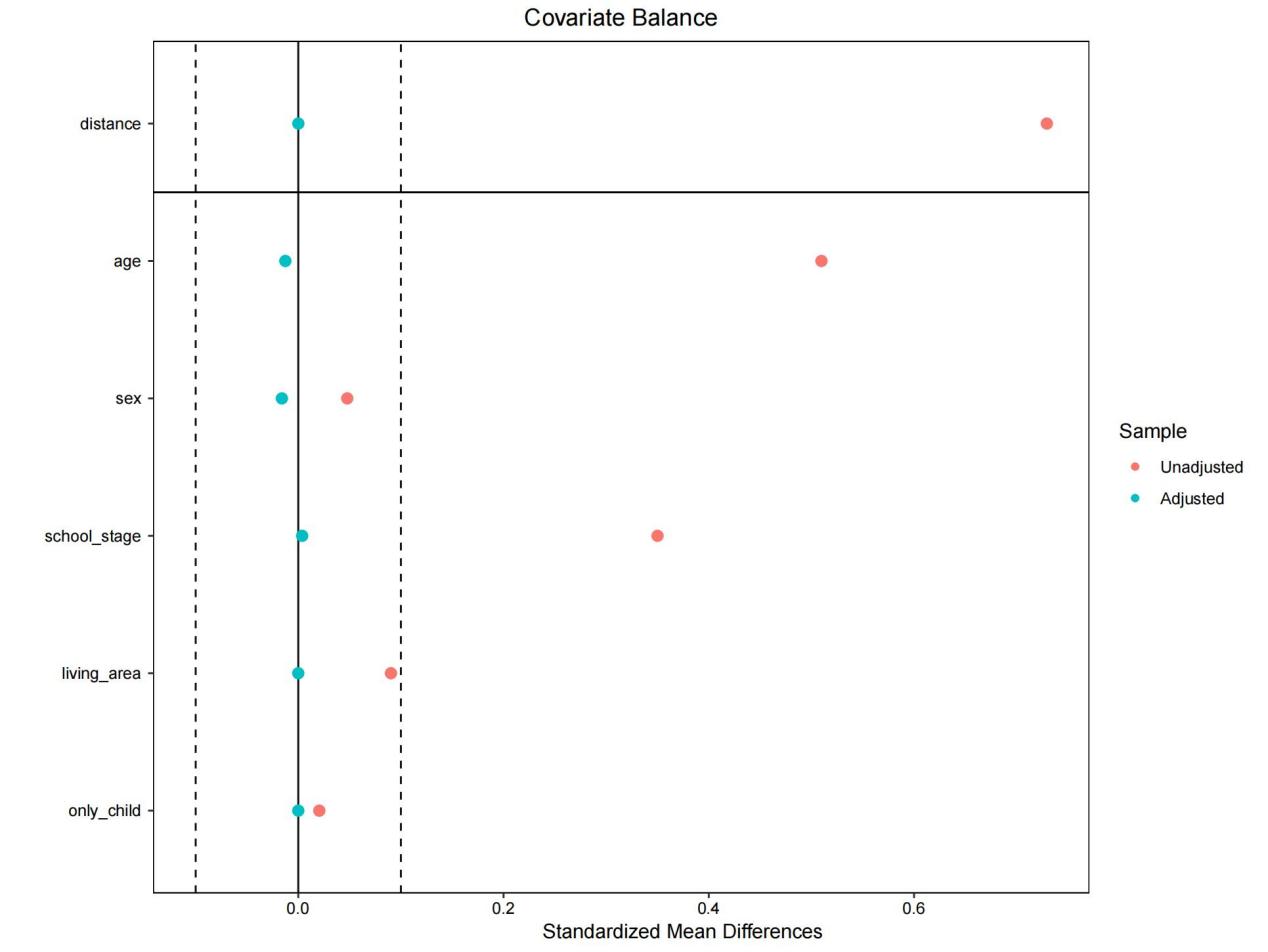
**

**
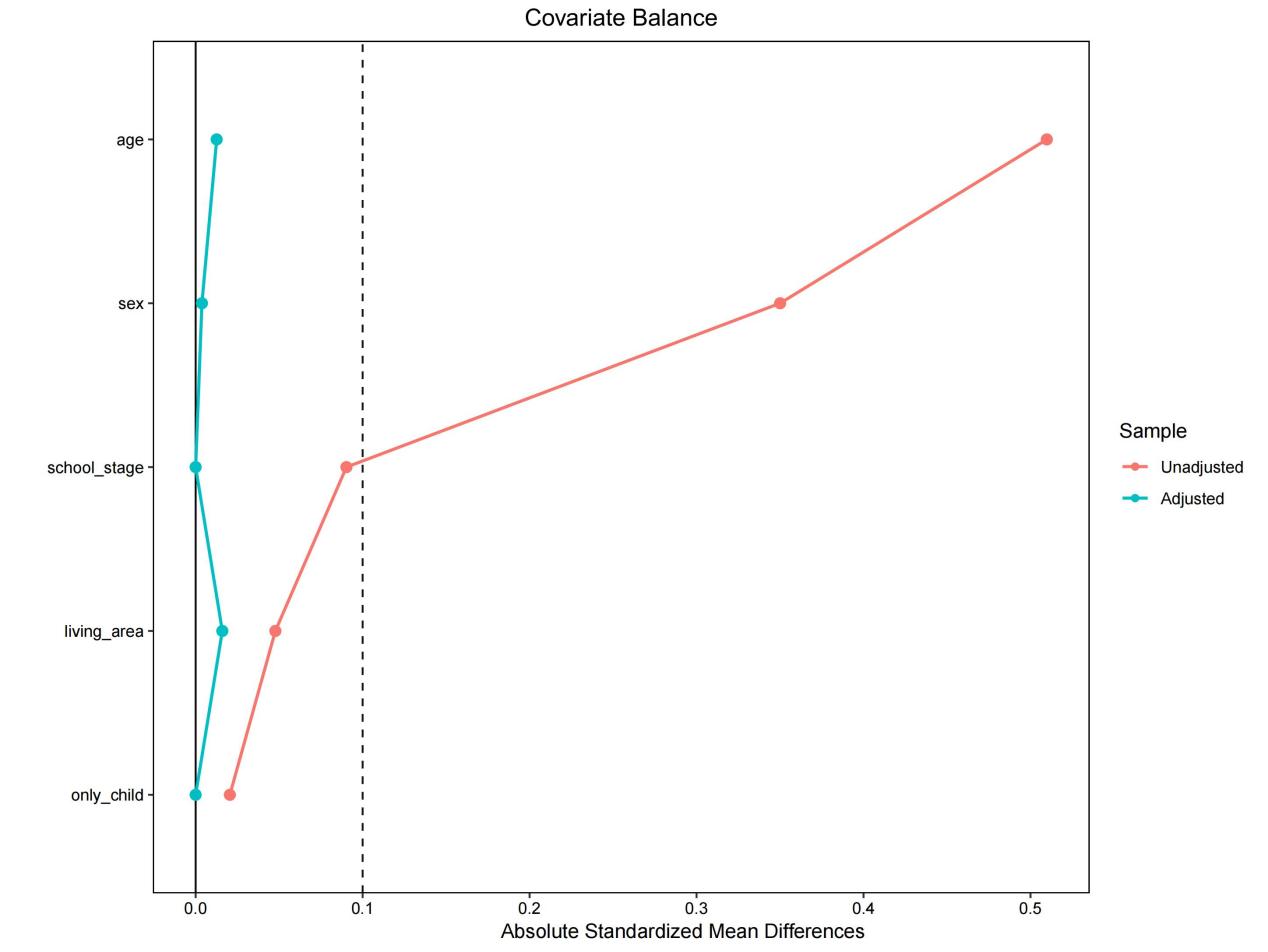
**

**Figure S1** Covariate distribution across SDG and NSDG before and after Propensity Score Matching. *Adjusted* means covariate after propensity score matching, SMD (all <0.1).


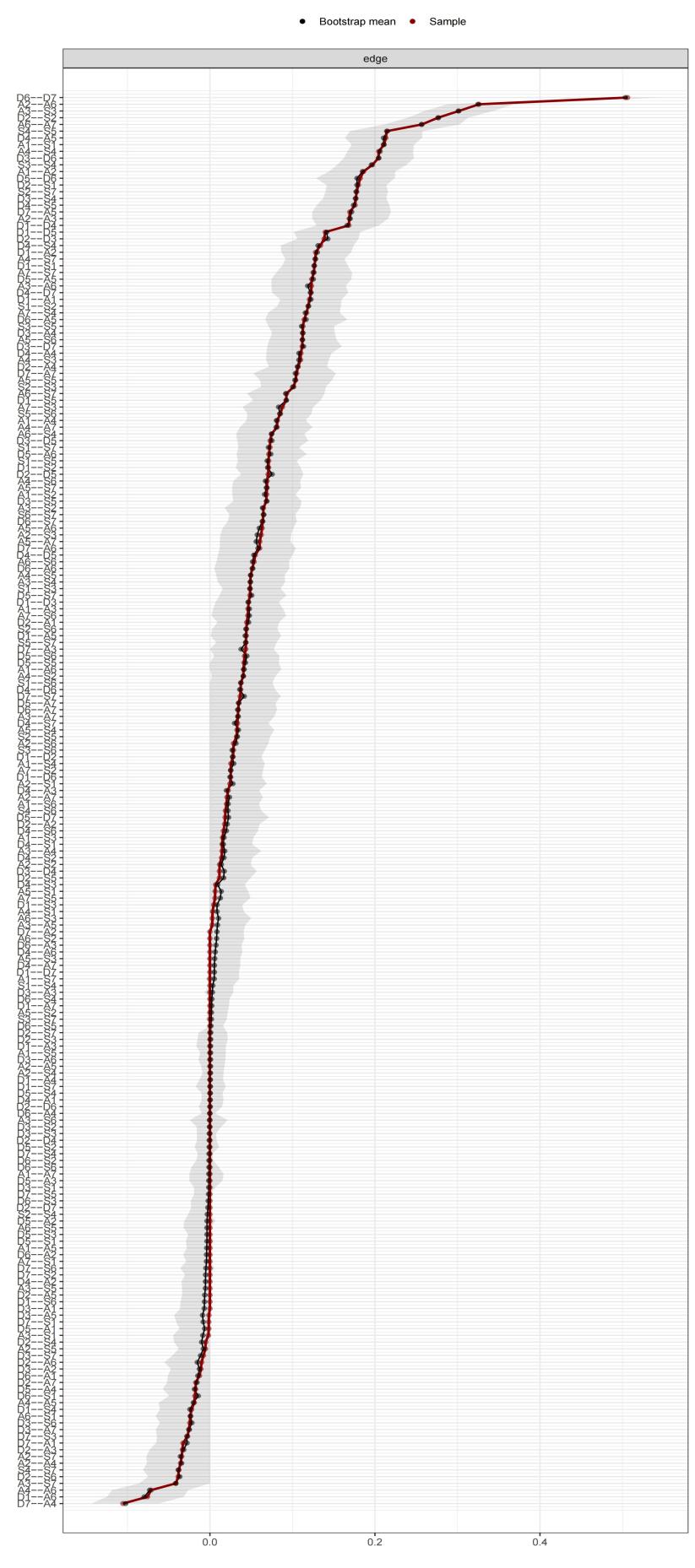


**Figure S2** Bootstrap 95% *CI* for edge weights in the network in SDG

**
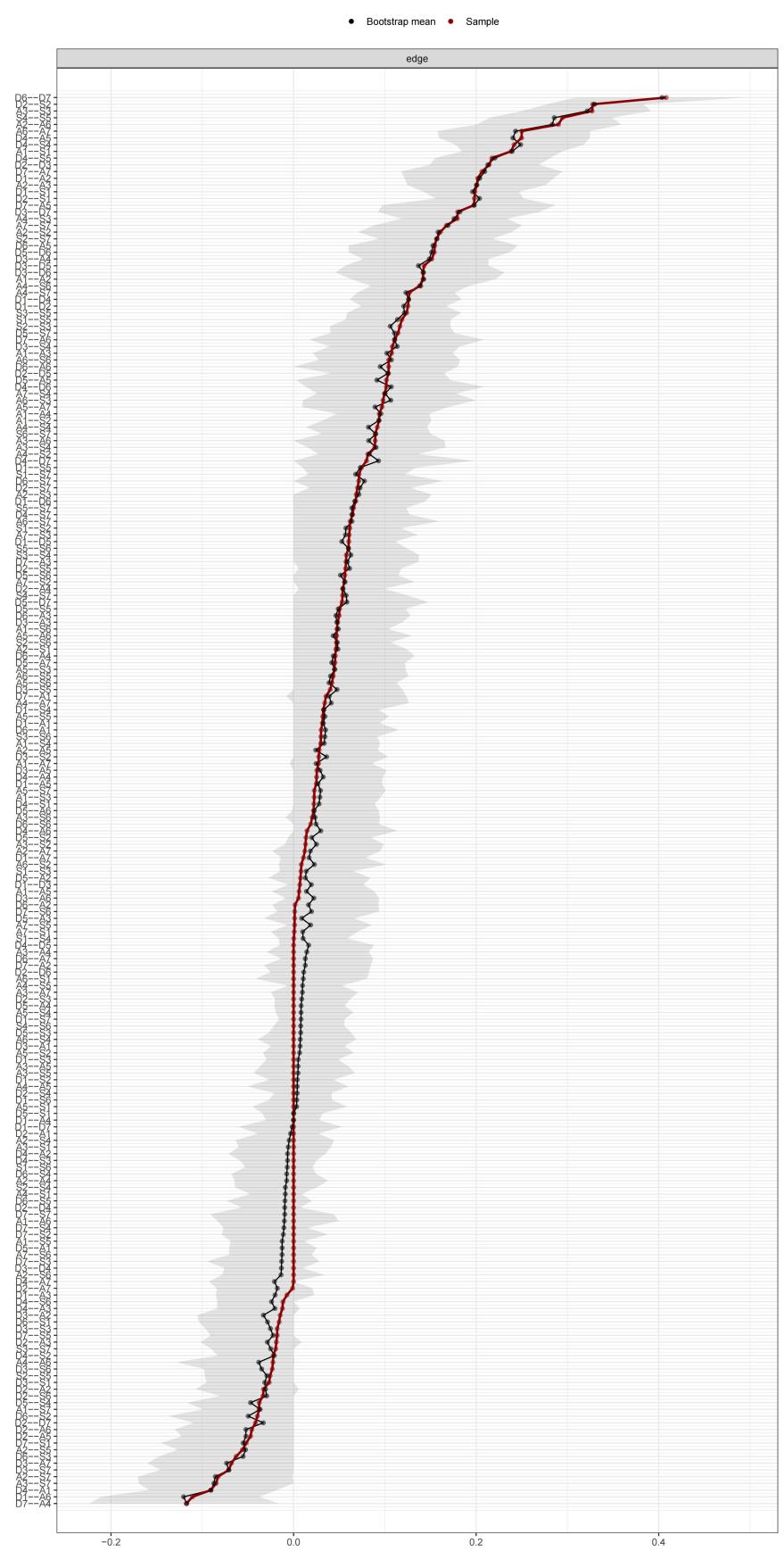
**

**Figure S3** Bootstrap 95% *CI* for edge weights in the network in NSDG

**
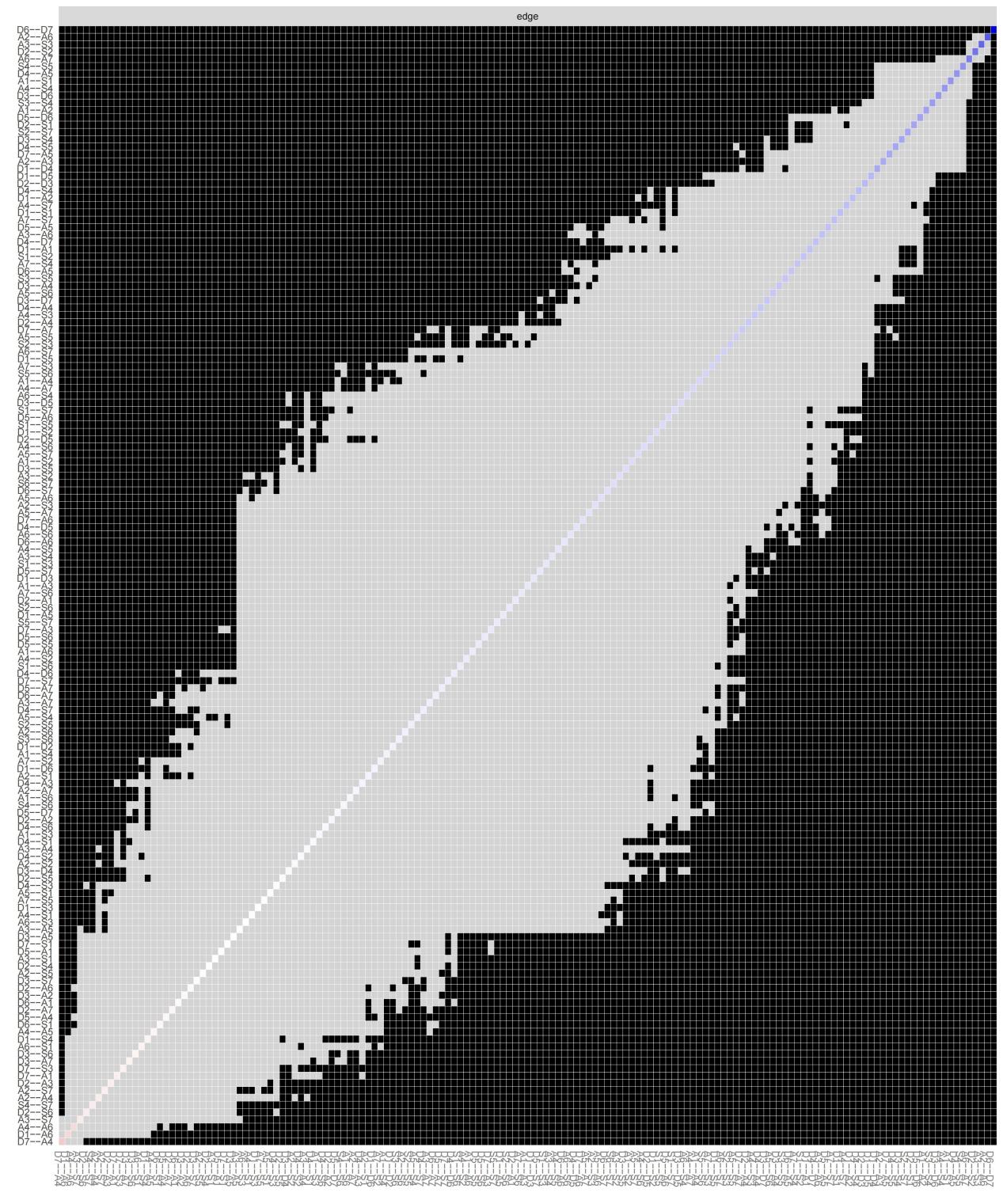
**

**Figure S4** Edge difference test in SDG

**
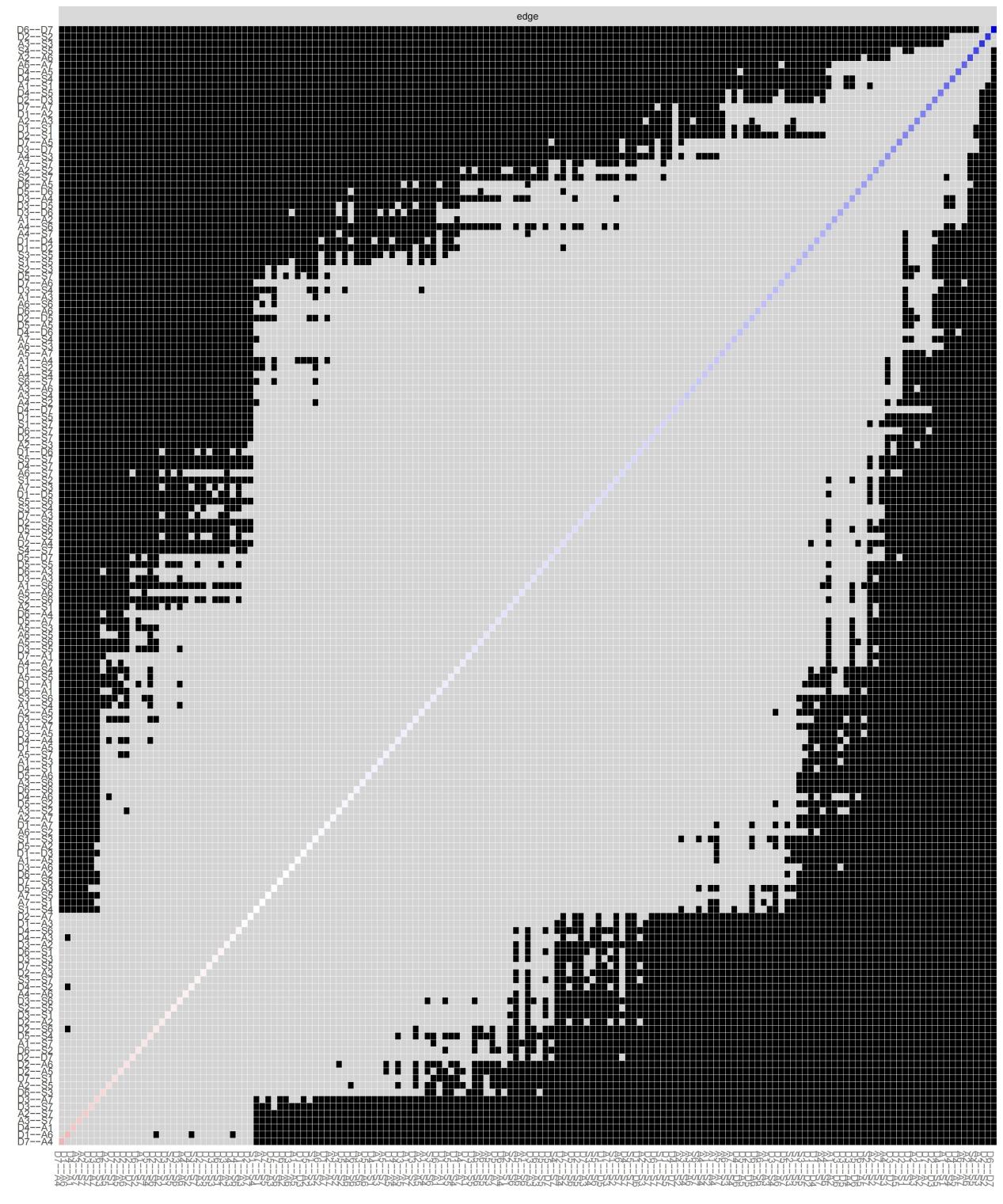
**

**Figure S5** Edge difference test in NSDG


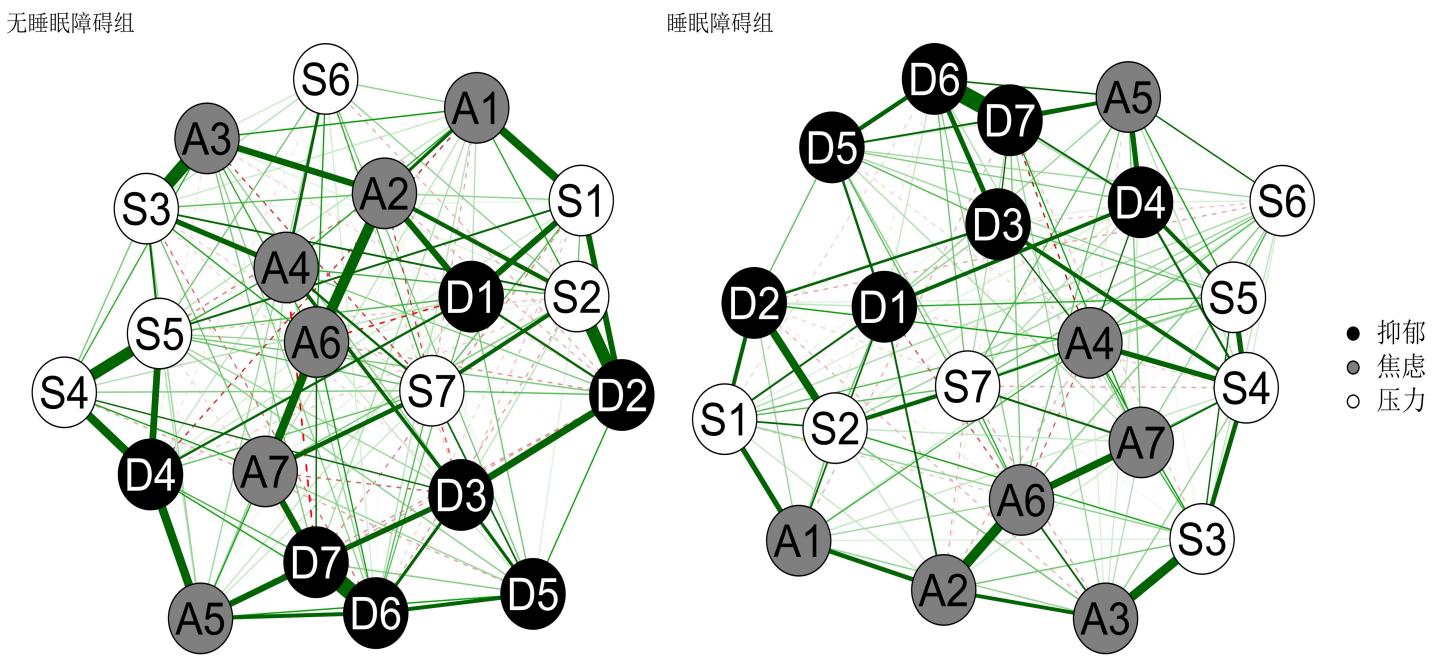


**Figure S6** Symptoms regularization partial correlations network in NSDG and SDG before matching


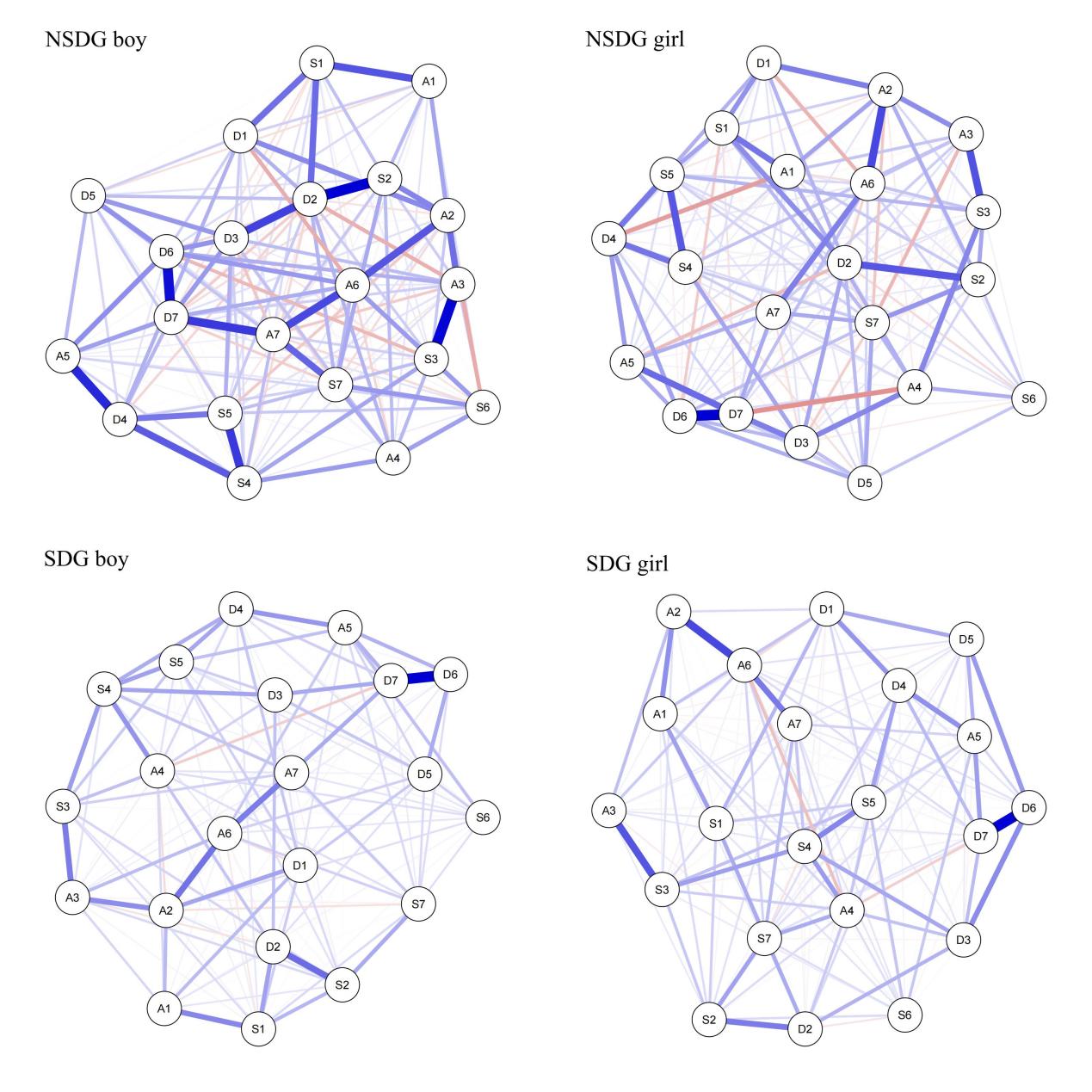


**Figure S7** Symptoms regularization partial correlations network in gender


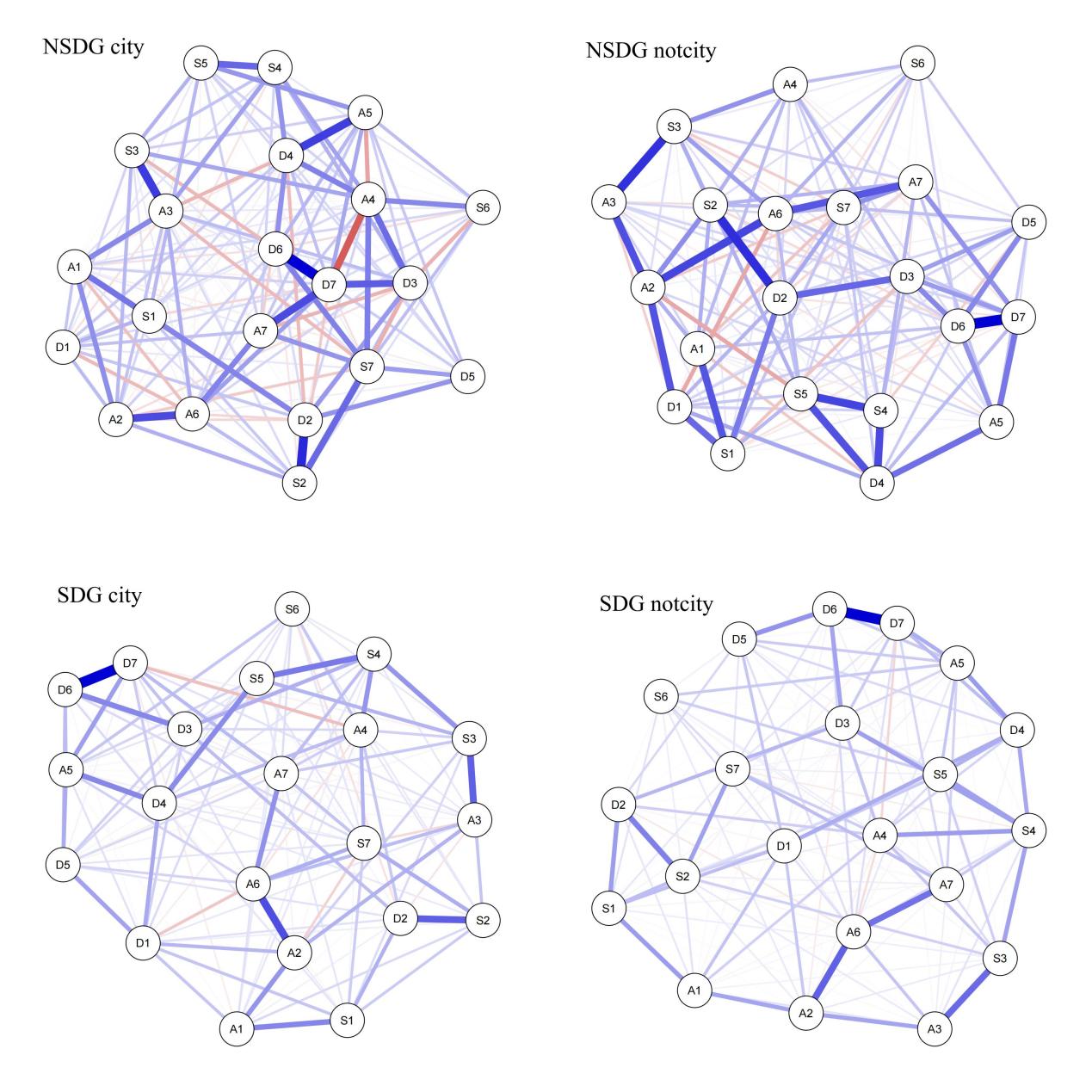
**Figure S8** Symptoms regularization partial correlations network in area


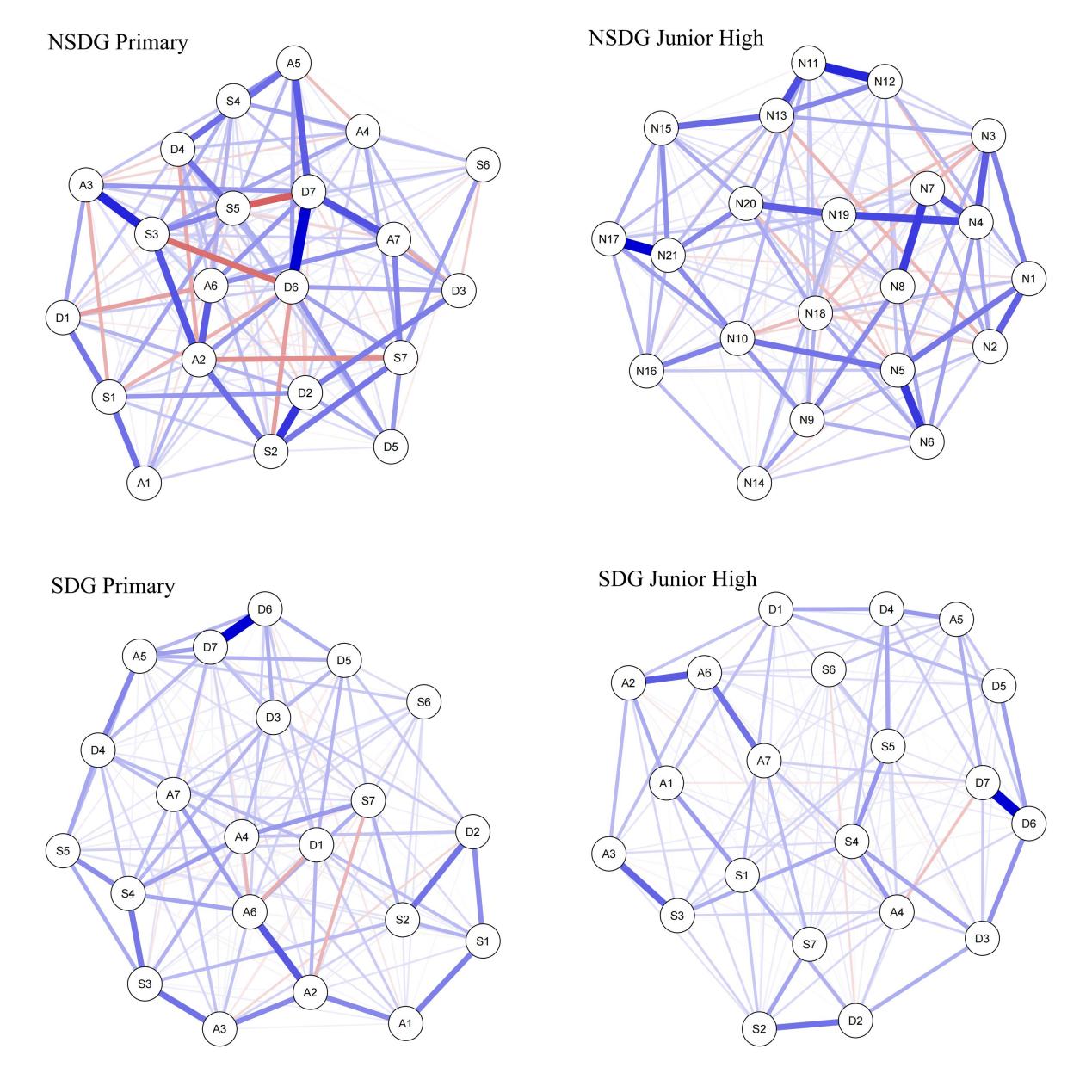
**Figure S9** Symptoms regularization partial correlations network in school stage


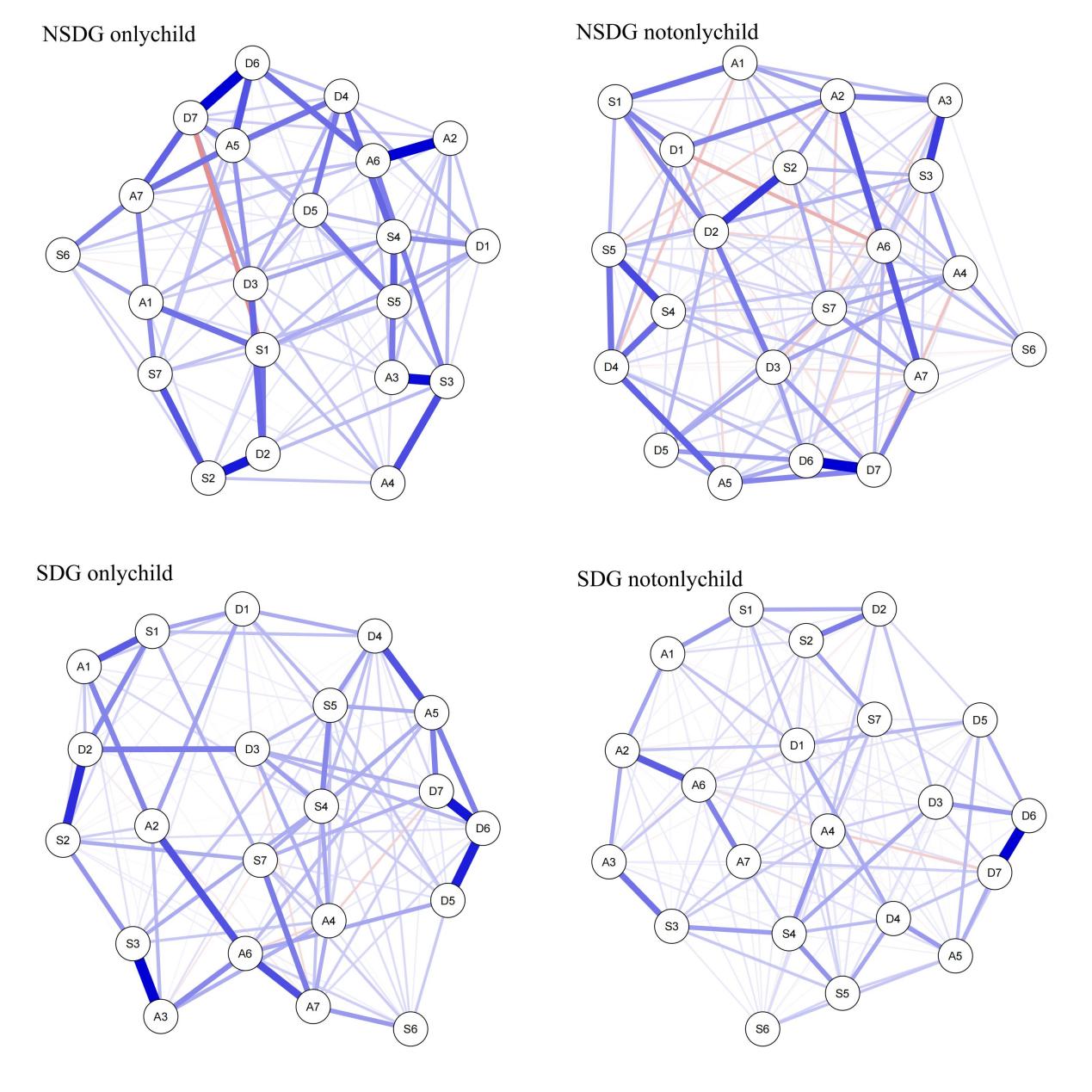
**Figure S10** Symptoms regularization partial correlations network in onlychild
